# Alzheimer’s Retinopathy: Seeing Disease in the Eyes

**DOI:** 10.3389/fnins.2020.00921

**Published:** 2020-09-08

**Authors:** Nazanin Mirzaei, Haoshen Shi, Mia Oviatt, Jonah Doustar, Altan Rentsendorj, Dieu-Trang Fuchs, Julia Sheyn, Keith L. Black, Yosef Koronyo, Maya Koronyo-Hamaoui

**Affiliations:** ^1^Department of Neurosurgery, Maxine Dunitz Neurosurgical Research Institute, Cedars-Sinai Medical Center, Los Angeles, CA, United States; ^2^Department of Biomedical Sciences, Cedars-Sinai Medical Center, Los Angeles, CA, United States

**Keywords:** neurological disease, neurodegenerative disorder, retina, ocular pathology, retinal imaging, optical imaging, amyloid-β plaques, hyperphosphorylated tau

## Abstract

The neurosensory retina emerges as a prominent site of Alzheimer’s disease (AD) pathology. As a CNS extension of the brain, the neuro retina is easily accessible for noninvasive, high-resolution imaging. Studies have shown that along with cognitive decline, patients with mild cognitive impairment (MCI) and AD often suffer from visual impairments, abnormal electroretinogram patterns, and circadian rhythm disturbances that can, at least in part, be attributed to retinal damage. Over a decade ago, our group identified the main pathological hallmark of AD, amyloid β-protein (Aβ) plaques, in the retina of patients including early-stage clinical cases. Subsequent histological, biochemical and *in vivo* retinal imaging studies in animal models and in humans corroborated these findings and further revealed other signs of AD neuropathology in the retina. Among these signs, hyperphosphorylated tau, neuronal degeneration, retinal thinning, vascular abnormalities and gliosis were documented. Further, linear correlations between the severity of retinal and brain Aβ concentrations and plaque pathology were described. More recently, extensive retinal pericyte loss along with vascular platelet-derived growth factor receptor-β deficiency were discovered in postmortem retinas of MCI and AD patients. This progressive loss was closely associated with increased retinal vascular amyloidosis and predicted cerebral amyloid angiopathy scores. These studies brought excitement to the field of retinal exploration in AD. Indeed, many questions still remain open, such as queries related to the temporal progression of AD-related pathology in the retina compared to the brain, the relations between retinal and cerebral changes and whether retinal signs can predict cognitive decline. The extent to which AD affects the retina, including the susceptibility of certain topographical regions and cell types, is currently under intense investigation. Advances in retinal amyloid imaging, hyperspectral imaging, optical coherence tomography, and OCT-angiography encourage the use of such modalities to achieve more accurate, patient- and user-friendly, noninvasive detection and monitoring of AD. In this review, we summarize the current status in the field while addressing the many unknowns regarding Alzheimer’s retinopathy.

## Introduction

Alzheimer’s disease (AD) is the most common age-related neurodegenerative disorder affecting over 50 million people worldwide (Hickman et al., 2016; Patterson, 2018). With no cure and limited options for early unambiguous and noninvasive diagnosis, this devastating and invariably fatal disease remains a major medical, sociological and economical challenge around the globe (Patterson, 2018; Alzheimer’s Association, 2019).

Patients with Alzheimer’s dementia typically exhibit symptoms of cognitive decline including disorientation, short-term memory loss, confusion, and socio-behavioral impairments. Beyond psycho-cognitive dysfunctions, these patients often experience visual abnormalities such as diminished color and contrast vision and narrowing of the visual field, as well as disruptions of circadian rhythms manifesting as sleep disturbances (Petersen et al., 1999; Perrin et al., 2009; Wang and Holtzman, 2020). Some of these visual dysfunctions and sleep irregularities have been documented early in the prodromal phase of AD in patients with mild cognitive impairment (MCI).

The neuropathological hallmarks of AD – amyloid β-protein (Aβ) plaques and neurofibrillary tangles (NFTs) comprised of hyperphosphorylated pTau protein – are well established and characterized in the brains of AD patients (Goedert et al., 1989; Hardy and Selkoe, 2002; Selkoe, 2004, 2008; Perrin et al., 2009; Goedert, 2015; Avila et al., 2016). These hallmark pathologies are hypothesized to induce and amplify inflammation and vascular abnormalities, drive synaptic and neuronal loss, and eventually lead to clinical AD-dementia (Choi et al., 2014; Ontiveros-Torres et al., 2016). The preclinical phase of AD-related pathological buildup is an insidious process which can take up to 20 years (Perrin et al., 2009; Bateman et al., 2012; Dubois et al., 2015; Bilgel et al., 2016; De Strooper and Karran, 2016; Dubois, 2018; Douglas and Scharre, 2019). Intervention during this preclinical stage, before definitive clinical symptoms appear and when synaptic and neuronal damage is still limited, should hold promise for increased therapeutic efficacy.

Notably, a recent research framework by the National Institute on Aging and Alzheimer’s Association (NIA-AA) classifies AD as compared with other neurodegenerative diseases based on molecular biomarker detection (Jack et al., 2018). The pathobiological phases of AD in living patients, irrespective of cognitive status, are determined on a continuum beginning with Alzheimer’s pathologic change and progressing to full AD by molecular biomarkers: Aβ deposition (A), pathologic tau (T), and neurodegeneration [AT(N)]. Accordingly, [N] is not specific to AD and the presence of (A) is necessary to define Alzheimer’s continuum (Jack et al., 2018; Gauthier and Rosa-Neto, 2019). Indeed, together with other genetic, longitudinal brain imaging, physiological, and pathological studies, this framework emphasizes that Aβ and tau pathologies are required to define AD. Moreover, as efforts to develop and evaluate other potential AD biomarkers intensify, including those measuring vascular changes, inflammation and synaptic loss (Chen et al., 2018; Baldacci et al., 2020), the AT(N) system remains flexible to incorporate novel biomarkers upon future availability.

In addition to the pathology described above, examination of postmortem AD brains demonstrated the existence and propagation of intra- and extracellular, soluble and synaptotoxic forms of Aβ oligomers (Selkoe, 2008; Meli et al., 2014; Li et al., 2020) and pTau assemblies (Takeda et al., 2015; Klein et al., 2019). Other key features of AD neuropathology include cerebral amyloid angiopathy (CAA) (Vinters, 1987) and neuroinflammation – the latter usually involving prolonged activation of microglia and astrocytes, release of pro-inflammatory cytokines and chemokines, as well as infiltration of peripheral immune cells [reviewed in Wyss-Coray (2006)]. In recent decades, cumulative evidence supporting the neuroprotective effects of certain immune cell types and inflammatory mediators have caused a historic shift in the view of neuroinflammation away from the common, merely detrimental one (Zuroff et al., 2017; Deczkowska et al., 2018; Schwartz et al., 2020). In this regard, studies showed that subtypes of bone marrow (BM)-isolated peripheral innate immune cells (e.g., BM-derived CD115^+^Ly6C^hi^CD45^hi^ monocytes) can be recruited to the diseased brain of AD-model mice. Interestingly, the recruited cells were shown to directly facilitate Aβ clearance, reduce chronic and detrimental inflammation including scar tissue proteins, and induce synaptogenesis and neurogenesis (Simard et al., 2006; Butovsky et al., 2007; Koronyo-Hamaoui et al., 2009; Lebson et al., 2010; Bernstein et al., 2014; Koronyo et al., 2015; Rentsendorj et al., 2018; Koronyo-Hamaoui et al., 2019; Li et al., 2020). These and other promising multi-targeted immunomodulatory strategies of harnessing peripheral immune cells to fight neurodegeneration are currently being developed and tested in various preclinical and clinical trials across the world (Frenkel et al., 2005; Butovsky et al., 2006; Bakalash et al., 2011; Kunis et al., 2013; Villeda et al., 2014; Koronyo et al., 2015; Baruch et al., 2016; Koronyo-Hamaoui et al., 2019; Rosenzweig et al., 2019; Li et al., 2020). Despite these advances, limitations persist due to the lack of readily available *in vivo* approaches to noninvasively and accurately monitor therapeutic efficacy, motivating scientists to search for new tools that can be widely deployed in the clinical setting.

Indeed, in recent years there has been tremendous progress in the development of AD diagnostic imaging biomarkers, including FDA-approved brain amyloid imaging via positron emission tomography (PET), as well as tau, surrogate markers of inflammation such as TSPO, and synaptic PET imaging (Rabinovici et al., 2007; Schilling et al., 2016; Chen et al., 2018; Edison et al., 2018; Narayanaswami et al., 2018; Chandra et al., 2019; Werry et al., 2019). In addition, structural, functional and metabolic brain imaging are instrumental in evaluating neuronal damage in patients (Johnson K.A. et al., 2012). Yet, most of these methods are not suitable for repeated population screening in the preclinical stages. They are either limited by the use of unsafe ionizing isotopes (radioactivity), high costs, low availability, and/or limited resolution or specificity (Perrin et al., 2009; Ono and Saji, 2011; Johnson J.K. et al., 2012; Johnson K.A. et al., 2012; Koronyo et al., 2012; Mathis et al., 2012; Doraiswamy et al., 2014; James et al., 2015; Kalia and Lang, 2015; Hart et al., 2016; Heurling et al., 2016; Tiepolt et al., 2016; Doustar et al., 2017; Hampel et al., 2018; Baldacci et al., 2020). As it relates to amyloid PET imaging, available tracers and modalities do not allow for detection of early small or soluble forms of Aβ accumulation (Lockhart et al., 2007; Ng et al., 2007; Johnson K.A. et al., 2012).

Milestone advances in the development and evaluation of plasma (Aβ_1–42_ and neurofilament light concentrations) and CSF biomarkers (Aβ_1–42_, total-tau, p-tau concentrations) have also revolutionized the prospect of early diagnosis for AD (Hampel et al., 2018; Baldacci et al., 2020). In general, both categories of fluid biomarkers have proven superior to the majority of currently available brain scanning tools with regards to sensitivity, accessibility and cost-effectiveness. Yet, CSF extraction remains an invasive approach and plasma markers are subject to interference from other bodily organs and peripheral metabolic processes, and therefore may not be able to solely represent the underlying neuropathological events occurring in the brain (Johnson K.A. et al., 2012). These challenges provide great incentive to explore other diagnostic methods for deployment in the general population, perhaps through another CNS tissue – the retina.

**FIGURE 1:**
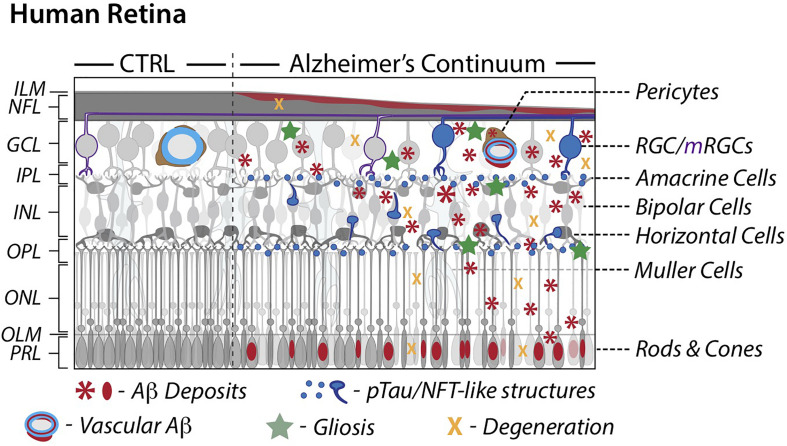
Schematic illustration of retinal pathology in AD patients. A comparison between cognitively normal control and AD retinas along a continuum. AD attributed retinal pathology includes accumulation of Aβ and phosphorylated-tau proteins, degeneration and subsequent thinning, and an inflammatory response. AD retinal vessels display substantial thinning and pericyte loss alongside Aβ protein deposition. Modified illustration from Advances in Retinal Imaging: Retinal Amyloid Imaging (Koronyo-Hamaoui et al., 2020) with permission from Springer Nature via Copyright Clearance Center. Aβ, Amyloid-β protein; CTRL, control; GCL, ganglion cell layer; ILM, inner limiting membrane; INL, inner nuclear layer; IPL, inner plexiform layer; mRGCs, melanopsin-containing retinal ganglion cells; NFL, nerve fiber layer; NFT, neurofibrillary tangles; OPL, outer plexiform layer; ONL, outer nuclear layer; OLM, outer limiting membrane; PRL, photoreceptor layer; RGCs, retinal ganglion cells; pTau, hyperphosphorylated tau.

Serving as the innermost layer of the eyeball, the main role of the retina in visual perception is the conversion of light signals into decodable neuronal impulses for transmission to the brain. It consists of the light-sensitive neurosensory retina and retinal pigment epithelium (RPE). Structurally, the neural retina contains several distinct layers: the innermost inner limiting membrane (ILM), nerve fiber layer (NFL), ganglion cell layer (GCL), inner plexiform layer (IPL), inner nuclear layer (INL), outer plexiform layer (OPL), outer nuclear layer (ONL), outer limiting membrane (OLM), and the outermost photoreceptor layer (PRL) (see left panel in Figure 1). The five types of neurons for visual perception in the retina are photoreceptors, bipolar cells, ganglion cells, horizontal cells, and amacrine cells. The two types of photoreceptor cells, rods and cones, are the primary light-sensing cells. Rods are responsible for sensing dim light, while cones provide color vision. Amacrine cells and bipolar cells are the two intermediate neurons that pass visual information to the ganglion cells, which then transmit signals to the brain. Similar to the brain, the retina also possesses different types of supporting glial cells: Müller cells, astrocytes, and microglia. Müller cells and astrocytes are the two types of macroglia in the retina and provide support to neurons, while microglial cells act like tissue macrophages. Blood is supplied to the retina via the central retinal artery, which enters the optic nerve and supports the inner neural retina, and via choroid blood vessels, which nourish the RPE and outer neural retina.

The retina is the only CNS tissue not shielded by bone, allowing for noninvasive imaging and providing a unique perspective into the brain. A developmental outgrowth of the embryonic diencephalon, the retina fittingly shares many structural and functional features with the brain including a blood barrier and populations of neurons and glial cells, which secrete proteins related to the amyloid cascade (e.g. BACE1, γ-secretase, ApoE, clusterin) (Morin et al., 1993; Purves and Fitzpatrick, 2001; Johnson et al., 2002; Byerly and Blackshaw, 2009; Maude et al., 2009; Cai et al., 2012; Li et al., 2016; Trost et al., 2016; Vecino et al., 2016). The retina is physically connected to the brain via axons of the optic nerve (Figure 1A), which facilitate vesicular transport of synthesized AβPP, potentially to and from retinal ganglion cells (RGCs) (Morin et al., 1993). Further, brain and retinal microvasculatures are morphologically and physiologically similar (Patton et al., 2005). Overall, the close relationship between these two CNS tissues and their anatomical sub-structures as well as the feasibility of noninvasive retinal imaging may provide a window into better understanding of processes in the CNS such as healthy aging and neurodegeneration.

Mounting evidence demonstrate AD-related retinal pathology in AD patients and animal models. Recent studies documented parallels between the brain and retinal pathology found both in AD patients and animal models (Koronyo-Hamaoui et al., 2011; Koronyo et al., 2012; Shi et al., 2014; Hart et al., 2016; Doustar et al., 2017; Koronyo et al., 2017; Koronyo-Hamaoui et al., 2020). While early examinations of postmortem eyes isolated from AD patients revealed loss of optic nerve integrity and RGC degeneration (Hinton et al., 1986; Blanks et al., 1989; Sadun and Bassi, 1990; Blanks et al., 1996a, b), it was not until 2010 that Koronyo-Hamaoui and colleagues were able to identify the existence of pathological hallmarks, Aβ deposits, in retinas isolated from these patients (Koronyo-Hamaoui et al., 2011). These findings were also true for MCI and other early-stage AD cases. Subsequent studies confirmed the original results and further identified pTau, characterized retinal plaque subtypes, as well as demonstrated neuronal degeneration and elevated levels of Aβ alloforms, astrogliosis and microgliosis (Alexandrov et al., 2011; Koronyo-Hamaoui et al., 2011; Koronyo et al., 2012, 2017; Schön et al., 2012; Tsai et al., 2014; Hart et al., 2016; La Morgia et al., 2016; den Haan et al., 2018; Hampel et al., 2018; Grimaldi et al., 2019; Schultz et al., 2020; Shi et al., 2020). In this review, we describe key pathological processes that were found in the AD retina (illustrated in Figure 1), with a focus on characteristic Aβ and tau accumulation and emerging retinal amyloid imaging modalities, which provide promise for advancing noninvasive methods for early disease diagnosis and monitoring.

## Retinal Aβ Pathology in Alzheimer’s Patients

**FIGURE 2:**
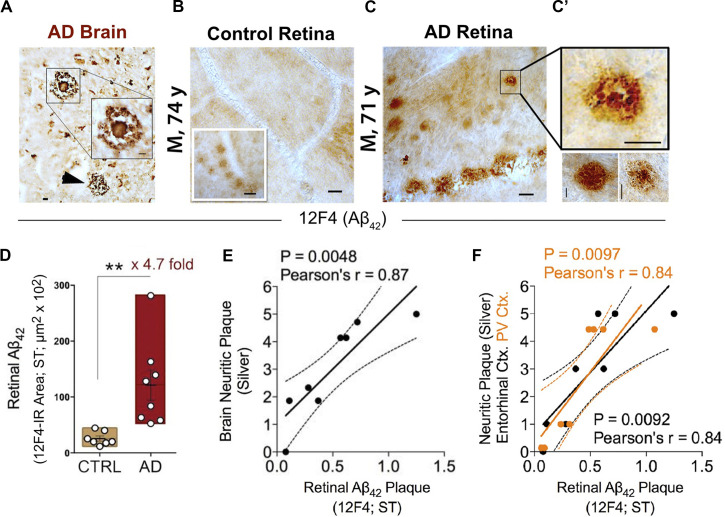
Increased retinal Aβ_42_ deposition correlates with cerebral amyloid plaque burden in Alzheimer’s patients. **(A)** Representative micrographs from an AD brain and **(B)** flat-mount retinas from a cognitively normal (CN) subject and **(C)** AD patient stained with anti-Aβ_42_ mAb (12F4). Although smaller in size, retinal Aβ plaques are similar in morphology to brain plaques. Scale bar: 20 μm. **(C)** High-magnification images reveal diffuse, compact, and “classical” mature plaque morphology of retinal Aβ aggregates. Scale bar: 10 μm. **(D)** Quantitation of retinal Aβ_42_ plaque burden, measured by 12F4 immunoreactive area, in AD patients (*n* = 8) and sex-/age-matched CN control subjects (*n* = 7). Data shown as group mean ± SEM. **P < 0.01, unpaired 2-tailed Student’s *t*-test. **(E,F)** Pearson’s correlation coefficient tests between retinal Aβ_42_ plaque load (12F4-immunoreactivity) and mean cerebral neuritic plaque burden (e; *r* = 0.87, *P* = 0.0048, *n* = 8; severity score of Gallyas silver staining) or regional plaque burden either in the entorhinal (**F**; black symbols; *r* = 0.84, *P* = 0.0092, *n* = 8) or primary visual cortex (**F**; orange symbols; *r* = 0.84, *P* = 0.0097, *n* = 8). Reproduced from Koronyo et al. (2017) with permission of ASCI via Copyright Clearance Center.

One decade ago, and more than a century following the identification of Aβ plaques in the postmortem brain of the first person diagnosed with AD, Auguste Deter, AD-specific pathological hallmarks were shown for the first time in the human retina (Koronyo-Hamaoui et al., 2011). In this original study, Aβ plaques were identified in all flatmount retinas isolated from 13 cases with definite and probable AD, as confirmed by both brain pathology and clinical reports (Figures 2A–C’). Retinal Aβ-plaque pathology in these patients was in stark contrast to minimal to no pathology found in the retina of age- and gender-matched cognitively normal individuals (Figures 2B–C’) (Koronyo-Hamaoui et al., 2011; La Morgia et al., 2016; Koronyo et al., 2017).

This and two subsequent studies on human cohorts of over 50 patients and control donor eyes, examining retinal flatmounts and cross-sections with Aβ-specific monoclonal antibodies (12F4, 11A5-B10, 6E10, 4G8), anti-Aβ dyes (i.e., Curcumin, Thioflavin-S, Congo-Red) and Gallyas silver stain, showed that all neuropathologically confirmed AD patients exhibited Aβ deposits in the retina (Koronyo-Hamaoui et al., 2011; La Morgia et al., 2016; Koronyo et al., 2017). Interestingly, through scanning of retinal flatmounts, the team discovered a non-uniform manifestation of Aβ deposits across the human retina. Plaques were more often detected in peripheral regions, especially in the superior and inferior quadrants (Koronyo et al., 2017).

A quantitative histological analysis of whole-mount retinas in a subset of confirmed AD patients compared to age- and sex-matched cognitively normal controls revealed a significant 4.7-fold increase in Aβ_42_-containing retinal plaque burden in patients (Figure 2D; Koronyo et al., 2017). Another group, using a sample of retinas isolated from 10 neuropathologically and clinically confirmed AD and 10 control patients, demonstrated a significant 2.7-fold increase in the number of retinal Aβ_42_ plaques that were also found to be larger in volume relative to deposits detected in normal control tissue (Grimaldi et al., 2019). Importantly, although retinal Aβ plaques are typically smaller in size compared to brain plaques, their burden in the retina significantly correlated with severity of plaque pathology in the brain (Figures 2E,F; Koronyo et al., 2017). In particular, retinal amyloid deposits were more strongly correlated with plaque burden in the primary visual cortex and the entorhinal cortex (Figure 2F; Koronyo et al., 2017).

**FIGURE 3:**
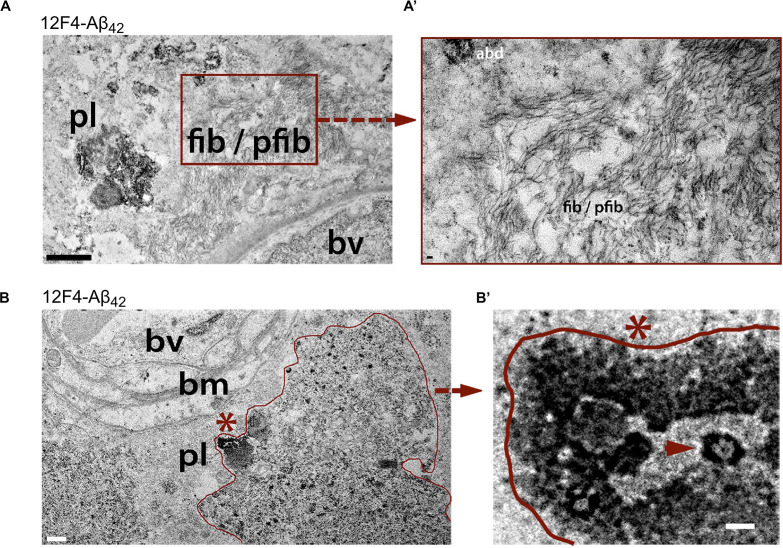
Ultrastructure of Aβ deposits in AD retina identified by transmission electron microscopy (TEM). **(A–B’)** Representative TEM images of retinal cross-sections from definite AD patients showing **(A)** ultrastructure of Aβ plaque (pl), fibrils (fib) and protofibrils (pfib) near a blood vessel (bv). Scale bar: 1 μm. **(A’)** High-magnification image showing Aβ fibrils, protofibrils and Aβ deposits (abd). Scale bar: 50 nm. **(B)** Aβ plaque-like deposits (pl; demarcated by red line), near basement membrane (bm) of a blood vessel (bv). Scale bar: 0.5 μm. **(B)** High magnification image of region marked by red asterisk in **(B)** showing dense Aβ deposition with structural similarity to annular oligomers (red arrowhead). Scale bar: 40 nm. All sections were prestained with anti-Aβ_42_ mAb (12F4) and peroxidase-based system and DAB substrate chromogen. Reproduced from Koronyo et al. (2017) with permission of ASCI via Copyright Clearance Center.

Transmission electron microscopy (TEM) analysis of (12F4^+^)Aβ_42_-positive immunoreactivity in retinal tissues from AD patients revealed the ultrastructure of Aβ in plaques, fibrils, protofibrils and annular oligomer-like forms (Figures 3A–B’; Koronyo et al., 2017). Gallyas silver stain further exposed the existence of retinal neuritic-like plaques. While marked increases in retinal Aβ pathology were noted in AD patients as compared with age-matched cognitively normal individuals, retinal plaques in patients frequently appeared in clusters and preferentially in the mid- and far-peripheral regions (La Morgia et al., 2016; Koronyo et al., 2017). These findings suggest that regional and geometric differences in plaque density should be considered when examining retinal tissue from patients. Moreover, the use of traditional histological techniques in retinal cross sections of limited regions could account for the few studies unable to consistently detect Aβ in the AD retina (Schön et al., 2012; Ho et al., 2014). This challenge emphasizes the necessity to standardize approaches for analyzing AD-related pathology across diverse topographical regions of the human retina. Indeed, following untraditional histological protocols developed by Koronyo and colleagues, three additional independent groups were able to detect Aβ deposits in the retina of confirmed AD patients (Tsai et al., 2014; den Haan et al., 2018; Grimaldi et al., 2019).

Prior examinations of normal aged eyes demonstrated Aβ immunoreactivity in the sub-retinal pigment epithelium (RPE) (Loffler et al., 1995) as well as toxic Aβ oligomers in drusen in the macular RPE of aged and AMD patients (Johnson et al., 2002; Anderson et al., 2004; Luibl et al., 2006). As it relates to AD, an early biochemical evaluation of Aβ_40_ and Aβ_42_ alloforms in retinal tissues of patients showed their existence in the human AD retina, albeit without comparing to levels in control retinas nor assessing correlation with respective brain levels (Alexandrov et al., 2011). Importantly, a recent study corroborated these findings of amyloidogenic Aβ_40_ and Aβ_42_ alloforms in the retina of AD patients (Schultz et al., 2020). The study measured Aβ_40_ and Aβ_42_ levels in retinal and hippocampal tissues of human cohorts with neurodegenerative diseases and compared between ApoE ε4 carriers and non-carriers (Schultz et al., 2020). Results from this study showed higher levels of retinal and hippocampal Aβ_40_ and Aβ_42_ in individuals with AD-related pathological changes and ApoE ε4 carriers. Further, levels of both alloforms in the retina correlated with their counterparts in the hippocampus, as well as with NFT and Aβ plaque burden severity (Schultz et al., 2020).

A recent histological study confirmed the presence of retinal plaques in 6 AD patients and 6 healthy controls, including finding similar sized 12F4^+^Aβ_42_-containing deposits, and gave additional insight into the spatial distribution and subtypes of Aβ aggregates in the human retina (den Haan et al., 2018). Aβ-positive immunoreactivity and deposits were found in various cell layers in postmortem retinas of AD patients, particularly the INL; deposits were found within horizontal, amacrine, and Müller cells (den Haan et al., 2018). Analyses of retinal pathology in AD donors indicated that Aβ deposits were more abundant in the inner retinal layers, concentrating in the NFL and GCL (Koronyo et al., 2017). Aβ_42_ was present in both fibrillar and proto-fibrillar forms, confirmed with TEM and Birefringence (apple-green) of Congo red-stained retinas under polarized light (Koronyo et al., 2017). More recently, Aβ_40_ was quantified and mapped in a larger cohort of postmortem human AD retinas (*n* = 47), showing significant increases in both retinal vascular and abluminal Aβ_40_ in AD patients as compared with matched controls (Shi et al., 2020). Retinal Aβ_40_ was especially abundant in the inner retinal layers of the central retina. Increased Aβ_40_ in the retina of AD patients as compared with cognitively normal individuals was further validated by biochemical ELISA analysis (Shi et al., 2020).

Overall, these growing studies confirm the presence of disease-associated Aβ species in the human retina and highlight the striking similarities between retinal and cerebral vulnerability to hallmark AD pathologies.

## Retinal Tauopathy in Alzheimer’s Patients

Another key characteristic sign of AD neuropathology that strongly reflects neuronal injury and cognitive decline is abnormal tau, specifically hyperphosphorylated tau and its inclusion in NFTs (Iqbal et al., 2009; Buckley et al., 2017; Hanseeuw et al., 2019). The physiological distribution pattern of total non-phosphorylated tau expression in the human retina was first described in 1995 (Loffler et al., 1995). According to this report and subsequent studies, tau is predominantly expressed in the inner retinal layers, most intensely along three distinct bands in the IPL, more diffusely in the OPL and somatodendritically in the INL (Loffler et al., 1995; Leger et al., 2011). Tau is also localized, albeit weakly, in other inner retinal layers such as the GCL and NFL as well as in photoreceptors of the human retina (Loffler et al., 1995; Leger et al., 2011).

**FIGURE 4:**
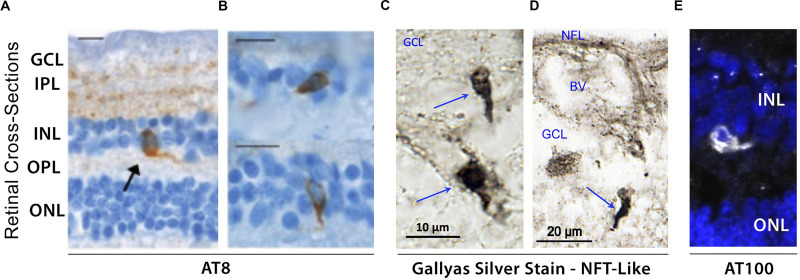
Evidence of hyperphosphorylated pTau inclusions in AD retina. **(A–E)** Representative micrograph images of retinal cross-sections from AD patients following **(A,B)** immunostaining with AT8 mAb against phospho-tau (Ser202, Thr205), revealing intracellular pTau aggregates in both inner and outer nuclear layers (INL and ONL), and plexiform layers (IPL and OPL). Scale bars: 10 μm; **(C,D)** Higher magnification images of inner retina stained with Gallyas silver showing neurofibrillary tangle (NFT)-like structures in ganglion cell layer (GCL). **(E)** Immuofluorescence staining of AT100 mAb against phospho-tau (Thr212, Ser214) showing punctate aggregates and intracellular inclusions in INL. Images and data of panels **(A,B)** are adapted from Schön et al. (2012). Images and data of panels c-d are reproduced from Koronyo et al. (2017) with permission of ASCI via Copyright Clearance Center.

Initial post-mortem examinations of late-stage AD retinas did not reveal neurofibrillary inclusions, neuritic plaques or amyloid angiopathy, despite histological observations of GCL degeneration, reduced NFL thickness and optic nerve axonal atrophy (Hinton et al., 1986; Blanks et al., 1989). While a limited number of studies have been unable to histopathologically detect abnormal tau accumulation in retinas of patients (Ho et al., 2014; Williams et al., 2017), the first evidence of disease-associated tau hyperphosphorylation in post-mortem retinas of confirmed AD cases was reported by Schön and colleagues in 2012 (Figures 4A,B; Schön et al., 2012). Results from this study were corroborated thereafter by other groups (den Haan et al., 2018; Grimaldi et al., 2019). Different pTau species, recognized by phosphorylation site-specific antibodies such as AT8 (pSer202, pThr205), AT100 (pThr212 and pSer214), and AT270 (pThr181), were primarily found in the inner retinal layers, particularly the plexiform layers, INL, and GCL of AD patients, thus closely mirroring the physiological expression pattern of normal tau (Schön et al., 2012; den Haan et al., 2018). Interestingly, a recent independent report on a quantitative histomorphometric analysis of post-mortem tissue revealed that these particular retinal layers undergo significant pathological atrophy in AD compared to non-demented control cases (Asanad et al., 2019b). Geometric analysis of postmortem retinal tissue from 6 control cases and 6 AD patients showed more intense AT8-immunoreactivity in superior than in medial retinal regions (den Haan et al., 2018). Qualitative observations from the same study also showed a positive gradient away from the optic nerve and toward the periphery. Despite the presence of pTau in all 6 AD retinas, no significant difference was found in retinal pTau area coverage between the two diagnostic groups, likely due to 2 outliers in control cases. Importantly, a novel and significant association was also found between retinal AT8 burden and cerebral amyloid plaque but not NFT severity (den Haan et al., 2018).

Hyperphosphorylation of tau has been shown to drive the formation of fibrillar tau inclusions and neurofibrillary tangles, leading to disruptions in axonal transport as well as metabolic and oxidative stress, and is closely associated with neuronal death (Iqbal et al., 2009). Although several histological staining approaches have been utilized to confirm the presence of fibrillar inclusions of pTau in human AD retinas, thus far many have been unsuccessful (Schön et al., 2012; den Haan et al., 2018). To date, only one study using Gallyas silver staining detected NFT-like structures in postmortem retinas of definitive AD patients (Figures 4C,D; Koronyo et al., 2017). As mentioned above, there is an apparent gap in results obtained from different groups, possibly driven by the lack of standardized experimental procedures. For instance, the same antibodies against pTau reported to produce negative results by one group (Schön et al., 2012) detected pTau staining patterns similar to AT8-immunoreactivity in a later study (den Haan et al., 2018). Still, by utilizing AT100 antibody, two independent groups detected similar patterns of pTau in postmortem retinas from AD patients (Figure 4E; den Haan et al., 2018; Grimaldi et al., 2019). Future investigations would therefore be necessary to confirm the presence or absence of disease-associated tau conformers in the retinas of AD patients.

Moreover, it is still unclear whether abnormal hyperphosphorylation of tau and/or formation of intracellular tangles have similar detrimental consequences in the retinas of patients as those observed in the brain. Analyses of retinal changes in AD transgenic mice support this putative association and will be discussed in the following sections. According to data from clinical studies and meta-analyses, levels of total tau and pTau in the CSF remain among the most reliable and sensitive biomarkers for both AD diagnosis and longitudinal monitoring of disease progression (Ibach et al., 2006; Welge et al., 2009; AlzBiomarker Database, 2018). Several lines of evidence indicate that CSF tau levels are significantly increased in AD patients (AlzBiomarker Database, 2018). Intriguingly, CSF total tau and pTau-181 concentrations were shown to correlate with retinal changes measured by fluorescent lifetime ophthalmoscopy, a technique suggested to detect the metabolic alterations of tissue represented by fluorescence decay of endogenous fluorophores (Jentsch et al., 2015). Nevertheless, the co-localization of pTau (den Haan et al., 2018; Grimaldi et al., 2019) and sites of neuronal loss in the retina (Koronyo et al., 2017; Asanad et al., 2019b; Grimaldi et al., 2019) are indicative of similar physiological vulnerabilities to pTau accumulation in both the retina and brain.

Retinal disorders such as glaucoma and age-related macular degeneration (AMD) share a number of common features with AD retinal pathology including progressive deposition of protein aggregates, reactive gliosis and pro-inflammatory responses, metabolic dysfunction, oxidative stress, and retinal atrophy (Quigley, 1999; Naskar et al., 2002; Gupta et al., 2006; Guo et al., 2007; Tezel, 2011). In 2008, examination of retinal tauopathy in surgically removed tissue from human glaucoma cases revealed intense localization of AT8-positive pTau in horizontal cells residing in the OPL (Ibach et al., 2006; Gupta et al., 2008). It is hypothesized that the lateral arrangement of horizontal cell processes may predispose them to retinal stretch injury caused by glaucoma-related elevated intraocular pressure (IOP). Although no association was found between high IOP and dementia (Cesareo et al., 2015), it remains to be seen whether similar changes occur due to AD-associated ocular abnormalities. However, increased retinal pTau accumulation and atrophy following injury or due to other neurodegenerative disorders reveal the vulnerability of this neural tissue compartment to structural, functional, and neuropathological abnormalities (Green et al., 2010; Leger et al., 2011; Kim et al., 2017; Satue et al., 2017; Behbehani et al., 2018; Kim et al., 2019).

Aging remains the principal risk factor in AD and is tightly associated with several visual impairments. To date, only two studies have investigated the relationship between aging and total tau expression in the human retina. An early semi-quantitative analysis found no difference between tau immunoreactivity in post-mortem retinas from young and old healthy subjects, although age ranges were not clearly outlined (Loffler et al., 1995). Another study, examining retinas from enucleated eyes of patients with prior history of ocular disorders, reported a positive correlation between aging and total tau levels in RGCs in a subset of patients, while unable to find evidence of pTau (Leger et al., 2011). Such reports further showcase the disparity in these findings and reiterate the need for replication of these studies. Notably, the effects of aging on the abnormal accumulation of pTau in retinas of healthy individuals and/or patients also remains unexplored.

As it relates to tau imaging in the retina, there are currently no live imaging tools to specifically detect tau aggregates in the human retina. Preliminary results from a study that utilized spectral domain optical coherence tomography (SD-OCT) and fundus autofluorescein (FAF) to visualize pathological tau aggregates in a cohort of PET-confirmed Alzheimer’s patients (Kayabasi, 2018) hints at the possibility of noninvasive live imaging and monitoring of neuropathological changes in the retina of MCI and AD patients; however, the specificity of the signal was not clear.

Overall, it is apparent that investigations of retinal Aβ pathology and its relationship with cerebral amyloid plaque burden in AD patients are mounting. However, our understanding of retinal pTau accumulation and associations with brain NFT severity is much more limited. To date, only a few groups have successfully detected tau hyperphosphorylation in post-mortem human retinas from AD patients, and only one study has shown an association between retinal pTau and cerebral amyloid load in a small number of AD patients. Therefore, there is an urgent need for systematic and quantitative analyses of retinal pTau in larger cohorts as well as assessment of both the spatiotemporal and pathomechanistic properties of AD-related tau species in the retina and their relationship with brain disease and cognition.

## Alzheimer’s Disease Hallmarks in the Retina of Animal Models

In agreement with the above findings in patients, the pathological hallmarks of AD were also described in numerous animal models of AD (see a summary in Table 1). Both the soluble and insoluble forms of Aβ were found in the retina of sporadic models and transgenic mice harboring familial AD (FAD) mutations (Ning et al., 2008; Dutescu et al., 2009; Liu et al., 2009; Perez et al., 2009; Koronyo-Hamaoui et al., 2011; Koronyo et al., 2012; Tsai et al., 2014; Du et al., 2015; Gupta et al., 2016; Hart et al., 2016; Habiba et al., 2020). Intriguingly, early manifestations of retinal Aβ plaques have also been detected prior to their occurrence in the brain (Koronyo-Hamaoui et al., 2011). Moreover, upon assessment of therapeutic response, researchers found that positive effects of immunotherapy on cerebral Aβ-plaque reduction were also reflected in the respective retinas in transgenic animal models of AD (ADtg) (Liu et al., 2009; Koronyo-Hamaoui et al., 2011; Koronyo et al., 2012; Yang et al., 2013; He et al., 2014; Gao et al., 2015; Parthasarathy et al., 2015). These studies illustrate the common retino-cerebral mechanisms of neuroprotection in response to therapy, which encourages the use of retinal imaging to noninvasively assess therapeutic efficacy in real time.

**TABLE 1:** Alzheimer’s pathological hallmarks in retinas of animal models.

Hallmarks	Animal species	Models and Genotypes	Retinal pathologies	References
APP or Aβ related	Drosophila	AβPP, dBACE-AβPPL, pGMR-Aβ_42_	Increased APP, Aβ, Aβ_42_ toxicity	Finelli et al., 2004; Greeve et al., 2004; Carmine-Simmen et al., 2009; Cutler et al., 2015
	Mouse	Tg2576, hTgAPP^tg/tg^, APP_SWE_/PS1_ΔE9_, APP_SWE_/PS1_M146L/L286V_, 3xTg, 5xFAD, Tg-SwDI	Increased APP, Aβ deposits, Aβ_42_, Aβ_40_, vascular Aβ, Aβ oligomers	Ning et al., 2008; Dutescu et al., 2009; Liu et al., 2009; Perez et al., 2009; Alexandrov et al., 2011; Koronyo-Hamaoui et al., 2011; Koronyo et al., 2012; Williams et al., 2013; Yang et al., 2013; Zhao et al., 2013; Edwards et al., 2014; He et al., 2014; Park et al., 2014; Gao et al., 2015; More and Vince, 2015; Parthasarathy et al., 2015; Pogue et al., 2015; Gupta et al., 2016; Oliveira-Souza et al., 2017; Criscuolo et al., 2018; Hadoux et al., 2019; Habiba et al., 2020; Sidiqi et al., 2020
	*O. degus*	Spontaneous	Increased APP, Aβ, Aβ oligomers	Inestrosa et al., 2005; Ardiles et al., 2012; Du et al., 2015
	Rat	TgF344-AD	Increased Aβ	Tsai et al., 2014
NFT or pTau related	Drosophila	hTau	Accumulation of pTau	Grammenoudi et al., 2006; Chouhan et al., 2016
	Mouse	Tg2576, APP_SWE_/PS1_ ΔE9_, APP_SWE_/PS1_M146L/L286V_, 3xTg, P301S, rTg4510	Accumulation of pTau, NFT	Liu et al., 2009; Schön et al., 2012; Yang et al., 2013; Zhao et al., 2013; Chiasseu et al., 2017; Grimaldi et al., 2018; Harrison et al., 2019
	*O. degus*	Spontaneous	Accumulation of pTau	Du et al., 2015; Chang et al., 2020

*APP, amyloid precursor protein; Aβ, amyloid-beta; pTau, phosphorylated Tau; NFT, neurofibrillary tangle; O. degus, Octodon Degus.*

ADtg animals typically express the transmembrane amyloid precursor protein (APP), the source of Aβ protein, in retinal neurons. Indeed, this protein has been found in the retinas of ADtg drosophila, various ADtg mice (Tg2576, hTgAPP^tg/tg^, APP_SWE_/PS1_ ΔE9_, and APP_SWE_/PS1_M146L/L286V_), and the naturally occurring sporadic rodent strain *Octodon degus* (*O. degus*) (Ning et al., 2008; Dutescu et al., 2009; Liu et al., 2009; Ardiles et al., 2012; Du et al., 2015). Retinal cytoplasmic APP was found to increase in ADtg models (Ning et al., 2008; Dutescu et al., 2009), but decrease in the sporadic *O. degus* with aging (Du et al., 2015). Ning and colleagues found APP immunoreactivity increased with age in cells of the INL and GCL – but not the ONL – as well as the neuropil of the IPL and OPL and the outer segments (OS) and RPE (Ning et al., 2008).

ADtg rodent models, including Tg2576, APP/PS1, 3xTg, 5xFAD mice, TgF344-AD rat, and *O. degus*, show cerebral accumulation of soluble and insoluble Aβ with age, corresponding to AD-like progression (Dutescu et al., 2009; Liu et al., 2009; Perez et al., 2009; Alexandrov et al., 2011; Koronyo-Hamaoui et al., 2011; Williams et al., 2013; Edwards et al., 2014; Park et al., 2014; Tsai et al., 2014; Du et al., 2015; More and Vince, 2015; Parthasarathy et al., 2015; Pogue et al., 2015). Alloforms of Aβ pathognomonic to AD (Aβ_40_ and particularly Aβ_42_) were found to be elevated in the retinas of ADtg rodents (Dutescu et al., 2009; Liu et al., 2009; Alexandrov et al., 2011; Williams et al., 2013; Park et al., 2014; Tsai et al., 2014; Du et al., 2015; Parthasarathy et al., 2015) and ADtg drosophila (Greeve et al., 2004; Carmine-Simmen et al., 2009). In addition, plaque and insoluble Aβ deposits were identified in retinas of Tg2576, APP_SWE_/PS1_ΔE9_, APP_SWE_/PS1_M146L/L286V_, 3xTg, and 5xFAD mice, and in *O. degus* (Ning et al., 2008; Dutescu et al., 2009; Liu et al., 2009; Perez et al., 2009; Alexandrov et al., 2011; Koronyo-Hamaoui et al., 2011; Koronyo et al., 2012; Williams et al., 2013; Yang et al., 2013; Zhao et al., 2013; Edwards et al., 2014; Du et al., 2015; More and Vince, 2015; Hadoux et al., 2019).

Tg2576 mouse retinas were cross-sectioned and analyzed for plaque pathology, which was found in approximately 85% of these transgenic mice but was absent in WT controls (Liu et al., 2009; Williams et al., 2013). Plaques were mostly found in the GCL, INL, and ONL (Liu et al., 2009; Williams et al., 2013). Yet, one study was unable to detect retinal Aβ pathology in a Tg2576 mouse with cerebral Aβ (Dutescu et al., 2009).

5xFAD mice present an aggressive model of amyloidosis, with several familial AD mutations that result in the overexpression of Aβ_42_. Studies in this model have demonstrated the presence of Aβ_42_ in ocular tissues including the retina as well as increases in Aβ_40_ in the RPE (Park et al., 2014; Parthasarathy et al., 2015; Hadoux et al., 2019). In the sporadic *O. degus* model of AD, Aβ deposition appears to be progressive, accumulating first in the GCL, NFL, INL and photoreceptors (Chang et al., 2020). Whole retinal histological examination via Aβ-specific staining revealed the most plaque burden in the central retina (Inestrosa et al., 2005; Du et al., 2015).

Importantly, Koronyo-Hamaoui et al. (2011) developed the first approach to visualize retinal Aβ deposits in live APP_SWE_/PS1_ΔE9_ mice using curcumin as a specific Aβ-labeling fluorophore and optical Micron rodent retinal imager (Phoenix Technology Group, LLC). The identity of Aβ deposits detected *in vivo* by curcumin was validated by subsequent *ex vivo* labeling of the respective whole-mount retinas using anti-Aβ monoclonal antibody. Notably, retinal plaques were observed prior to detection in the corresponding brains (Koronyo-Hamaoui et al., 2011). In addition, following intravenous injection of curcumin, flatmount retinas from 2.5-month-old mice were isolated and stained with 4G8 monoclonal antibody, further confirming that the curcumin spots are the same Aβ-specific deposits (Koronyo-Hamaoui et al., 2011). A recent study by Sidiqi and colleagues corroborated the previous reports (Koronyo-Hamaoui et al., 2011; Koronyo et al., 2012, 2017) and demonstrated via retinal curcumin imaging in APP_SWE_/PS1_ΔE9_ mice the accumulation of retinal Aβ plaques with disease progression, which positively correlated with Aβ load in the brain (Sidiqi et al., 2020). Cross-sectional analysis of adult APP/PS1 ADtg mice retinas revealed Aβ deposits in the innermost layers as well as in the choroid and surrounding scleral tissue; WT controls showed only minimal to no plaque deposition (Koronyo-Hamaoui et al., 2011). Studies using the same ADtg model and Tg344F-AD rats (with the same double transgenes) also validated these findings (Ning et al., 2008; Liu et al., 2009; Perez et al., 2009; Tsai et al., 2014).

The initial discovery of tau in the adult mammalian retina dates back to 1988. In this early study, tau was predominantly found in horizontal cells residing in the OPL of adult rat retinas (Tucker and Matus, 1988). On the other hand, disease-associated pTau species were detected somatodendritically in RGCs of a commonly studied double transgenic mouse model of AD, known as the APP_SWE_/PS1_ΔE9_ mouse (Yang et al., 2013), and in the GCL to ONL of Tg2576 ADtg mice (Liu et al., 2009). In line with these observations, intracellular aggregates of pTau have also been detected in retinas of the APP_SWE_/PS1_M146L/L286V_ mouse model of AD (Zhao et al., 2013). Further, recent analyses of young, pre-symptomatic 3xTg mouse retinas showed retinal Aβ plaques and tau tangles in the RGC layer (Grimaldi et al., 2018).

In the South American rodent *O. degus*, previously reported to develop several spontaneous AD-like pathologies without genetic manipulation, elevated levels of pTau were primarily detected in the GCL to NFL regions of the retina in both adult and aged animals (Du et al., 2015). In a subsequent study by the same group, early punctate AT8 immunoreactivity in the IPL was reported in young *degus*, compared with the denser expression in IPL to NFL of juvenile and adult animals (Chang et al., 2020). Interestingly, p(tau)-positive aggregates both appeared and propagated to other inner retinal layers earlier than Aβ deposits in these animals (Chang et al., 2020). Similarly, retinal accumulation of total tau and epitope-specific hyperphosphorylation were also reported to precede onset of behavioral deficits and brain tauopathy as early as 3 months of age in the 3xTg mouse model of AD (Chiasseu et al., 2017).

Several live imaging modalities have been developed and optimized for detection of proteinaceous aggregates and metabolic hyperspectral mapping of retinal tissue in rodent models of AD (Koronyo-Hamaoui et al., 2011; Koronyo et al., 2012, 2017; Schön et al., 2012; More and Vince, 2015; Sidiqi et al., 2020). Using longitudinal scanning laser ophthalmology, Schön et al. visualized and monitored pTau-containing RGCs in the P301S mouse model of tauopathy from 2 to 6.5 months of age and found a steady growth in pTau-positive cell counts (Schön et al., 2012).

In the rTg(tauP301L)4510 tauopathy mouse model of frontotemporal dementia (FTD), accumulation of both tau and pTau have been observed in RGCs as well as the IPL and INL (Harrison et al., 2019). These areas are associated with reduced neuronal density and optic nerve degeneration in these mice (Harrison et al., 2019). In an experimental rat model of optic nerve crush, injury-induced impaired autophagy was followed by an increase in hyperphosphorylated tau (pSer396), which co-localized with apoptotic markers in dying RGCs (Oku et al., 2019). Silencing the tau gene exerted neuroprotective effects in this model, indicating that tauopathy following retinal injury similar to that observed in the brain plays an essential role in neuronal atrophy (Oku et al., 2019).

AD-related tauopathies have also been investigated in a limited number of non-murine animal models of aging or AD. Selective expression of normal human tau in adult drosophila retina leads to progressive loss of ERG responses without a considerable effect on retinal structure or neuronal density, although TEM analysis later revealed signs of tau-induced retinal synaptotoxicity and abnormal photoreceptor morphology (Chouhan et al., 2016). In the same study, human Aβ expression in a separate group of flies caused an age-dependent loss of retinal neurons without altering ERG signals (Chouhan et al., 2016). In the UAS-Gal4 drosophila model of AD, species of pTau, phosphorylated at different epitopes to varying degrees and in a cell type-specific manner, have also been detected in both the retina and brain (Grammenoudi et al., 2006). In a small number of young and aged primates, total tau expression in the outer retina was observed in the OPL, ONL and inner segment of photoreceptors, whereas AT8-positive pTau was localized predominantly in the OPL and cones (Aboelnour et al., 2017). In older primates, pTau staining in retinal cones also appeared stronger compared with younger animals (Aboelnour et al., 2017). Altogether, it is apparent that tau expression and pTau accumulation may vary across several species commonly used to model human AD, and future studies should aim to elucidate these differences.

Together with these positive findings, it is important to note that the expression pattern of both APP and tau transgenes in the brain and retina of animal models of AD should not be misinterpreted as a precise reflection of the human disease. The layer and cellular burden of pathological Aβ and pTau aggregates is most likely governed by the promoter-driven expression of their corresponding transgenes. For instance, expression of the aggregate-prone mutant tau species in the P301S tauopathy mouse model is driven by the murine Thy1 promoter, which leads to the development of pathology in selected CNS cell types and consequently specific regions (Allen et al., 2002). In the retina, Thy1 is uniquely expressed by RGCs (Schmid et al., 1995) and therefore the intracellular aggregation of hyperphosphorylated tau in these cells and their nerve fibers is expected. This may underlie the common disparity in findings from stereological studies attempting to characterize regional and cellular susceptibility to AD pathology in CNS tissue from human versus animal models. Recent developments as well as ongoing efforts to fully characterize complete gene replacement animal models will be invaluable in addressing such limitations in several fields.

## Retinal Degeneration in Alzheimer’s Disease

In agreement with the observed distribution of retinal Aβ deposits in AD patients, reports have indicated NFL thinning and RGC degeneration in the GCL chiefly within the superior quadrants of the retina (Blanks et al., 1989, 1996b; Berisha et al., 2007; Lu et al., 2010; Liu et al., 2015; La Morgia et al., 2016; Asanad et al., 2019b; Grimaldi et al., 2019). *In vivo* OCT imaging of these patient retinas revealed degeneration in multiple retinal layers (La Morgia et al., 2017). Reduced macular thickness and volume, measured by OCT, was also found to correlate with cognitive impairment in AD patients (Iseri et al., 2006). The first study that evaluated melanopsin-containing retinal ganglion cells (mRGCs), photoreceptors known to drive circadian photoentrainment, in the postmortem retina of AD patients described a significant mRGC degeneration reflected in reduction of both dendritic density and cell number (La Morgia et al., 2017). Notably, dendrite loss and cell death were closely linked to Aβ pathology and were colocalized at sites of Aβ deposits (La Morgia et al., 2016).

**FIGURE 5:**
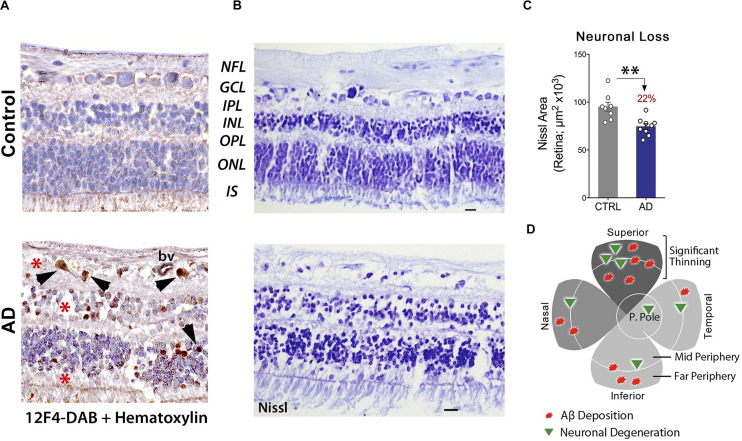
Increased Aβ_42_-associated neuronal loss in post-mortem retinas of AD patients. **(A)** Representative images of post-mortem retinal cross-sections from CN and AD cases, immunostained with anti-Aβ_42_ mAb (12F4) and labeled with peroxidase-based DAB (brown) and hematoxylin counterstain (violet). Intracellular Aβ_42_-inclusions are observed in GCL, INL and ONL of AD retina. Scale bars: 20 μm. **(B)** Nissl staining of retinal cross-sections from a control subject and AD patient, revealing cellular and retinal layer alterations in AD. **(C)** Quantitation of neuronal Nissl-positive total area in retinas of AD patients (*n* = 9) compared to age-/sex-matched CN control subjects (*n* = 8). Data shown as group mean ± SEM. **P < 0.01, unpaired 2-tailed Student’s *t*-test **(D)** Qualitative geometric map of increased retinal Aβ deposits (red shapes) and neuronal loss (green triangles) in AD patients. Panels a-c are reproduced from Koronyo et al. (2017) with permission of ASCI via Copyright Clearance Center. Panel d is adapted from Hart et al. (2016) under terms of the Creative Commons Attribution 4.0 International License (http://creativecommons.org/licenses/by/4.0/).

These findings were corroborated in another study, which indicated a substantial loss of retinal cells at sites of Aβ deposition (Figures 5A–D). When compared with age-matched cognitively normal controls, 22-29% retinal neuronal degeneration was detected by Nissl staining (n = 17 subjects) in the GCL, INL, and ONL of AD patients (Figures 5B,C; Koronyo et al., 2017). More recently, Asanad and colleagues analyzed retinal atrophy morphometrically in the superior quadrants of postmortem retinas from 8 AD and 11 age-matched controls (Asanad et al., 2019b). Measurements were acquired along a distance of 4mm from the optic nerve on the supero-temporal (reaching the macular region) and supero-nasal sides. Significant retinal thinning was revealed in the NFL (∼40%), GCL (35%), IPL (∼20%), and both nuclear layers (25%) in AD patients. In the supero-temporal region, NFL thinning was more pronounced closer to the optic nerve, whereas the other retinal layers showed prominent thinning closer to the macula. However, all analyzed retinal layers showed consistent thinning throughout the supero-nasal retinal quadrant (Asanad et al., 2019b). Beyond these changes, several studies have demonstrated accumulation of Aβ in the lens of AD patients (Goldstein et al., 2003; Tian et al., 2014) as well as reductions in the choroid coat – the vascular layer of the eye – and offered insights into the retinal choroid as an oculovascular biomarker for Alzheimer’s disease (Tsai et al., 2014; Asanad et al., 2019a).

These findings are further supported by animal models of AD (Frederikse and Ren, 2002; Yang et al., 2013; Park et al., 2014; Tsai et al., 2014). In the hTgAPP^tg/tg^ mouse lens, markers of degeneration included nuclear disorganization, organelle loss, cellular swelling, and shape irregularity (Frederikse and Ren, 2002). In the 5xFAD mouse, Aβ deposits in the retinal pigment epithelium, hypopigmentation, large vacuoles, and Bruch membrane thickening with Drusen-like deposits were observed (Park et al., 2014). Hypertrophy along with choroid changes were also detected in the TgF344-AD rat (Tsai et al., 2014). Further, in the APP_SWE_/PS1_ΔE9_ mouse, RGC density was lower than in WT controls (Gao et al., 2015; Gupta et al., 2016) and amacrine cell apoptosis was noted (Gao et al., 2015).

A recent finding in 3xTg mice indicated colocalization of both retinal tau tangles and Aβ plaques with neurodegeneration in the GCL (Grimaldi et al., 2018). In the same mouse model, tau accumulation was associated with retinal neural dysfunction, as measured by deficits in anterograde axonal transport (Chiasseu et al., 2017).

Altogether, observations of retinal atrophy in layers recognized as sites of proteinaceous deposition in both AD patients and animal models of the disease illustrate the vulnerability of retinal cell types to pathological processes traditionally associated with the cerebral disease. In addition, these findings further support the suitability of retinal thinning, in combination with more specific markers, as a diagnostic and monitoring tool for AD.

## Retinal Vascular Pathology in Alzheimer’s Disease

Cerebral amyloid angiopathy is defined as a cerebrovascular disease characterized by intense deposition of Aβ in the walls of cerebral arteries, arterioles, and capillaries, among other vascular damage, and is commonly found in the brains of AD patients (DeSimone et al., 2017). Aβ in CAA primarily consists of Aβ_40_ (Roher et al., 1993; Gravina et al., 1995). In macrovasculature, CAA is composed of Aβ deposition in tunica media and adventitia of leptomeningeal and cerebral parenchymal arteries (Tian et al., 2004). The Aβ in CAA will eventually affect all vascular layers and result in degeneration of smooth muscle cells (Keable et al., 2016). CAA is prevalent in the elderly who have developed lobar cerebral hemorrhage (ICH) (Keable et al., 2016). Although CAA remains a distinct clinical entity from dementia, previous studies have demonstrated that over 85% of AD patients have of CAA of varying severity (Arvanitakis et al., 2011; Viswanathan and Greenberg, 2011). The Aβ deposited in the vascular walls triggers several ischemia-induced pathogenic molecular pathways, such as oxidative stress, inflammation and increased blood-brain barrier (BBB) permeability, leading to further hemorrhagic complications (Ghiso et al., 2010). A study based on parametric analysis of neuropathological data from the National Alzheimer’s Coordinating Centers’ dataset suggested that CAA was facilitating early stage dementia and the transition to moderate dementia (Vidoni et al., 2016). In severe CAA cases, Aβ deposition is usually followed by microaneurysms in cerebral blood vessels (Vonsattel et al., 1991), a vascular pathology shared by retinal vascular diseases such as diabetic retinopathy, which can be easily examined by fundoscopy (Friberg et al., 1987; Hellstedt et al., 1996).

As the retina is a developmental outgrowth of the diencephalon (Purves, 2001; Erskine and Herrera, 2014), it shares many vascular morphological and physiological features with cerebral vessels (Patton et al., 2005; Crair and Mason, 2016). For instance, the retina has a highly selective blood retinal barrier (BRB), with many similar structural and functional properties as the BBB, which modulates the influx of ions, proteins and water, as well as limits the infiltration of circulating immune cells (Cunha-Vaz et al., 2011). The recent identification of an ocular glymphatic drainage system in rodents, clearing fluids and metabolites such as Aβ from the retina and vitreous via an aquaporin-4 (AQP4)-dependent pathway, is a sign of yet another structural and physiological similarity shared by the brain and retina (Wang et al., 2020). The same group also demonstrated that this clearance route may be impaired in ocular conditions associated with retinal damage such as glaucoma (Wang et al., 2020). Whether similar glymphatic drainage occurs in the human eye and the extent to which disruptions in this process contribute to pathological changes in neurological diseases such as AD remain to be investigated.

Numerous reports have broadly described vascular dysfunctions in the AD retina, including increased tortuosity, narrowed veins, decreased blood flow, microvascular network damage, and compromised branching complexity (Berisha et al., 2007; Frost et al., 2010, 2013; Cheung et al., 2014; Feke et al., 2015; Williams et al., 2015; Einarsdottir et al., 2016; Abbasi, 2017; Cabrera DeBuc et al., 2018; O’Bryhim et al., 2018), similar to earlier findings in the brain (Smith et al., 1999; Bookheimer et al., 2000; Kimbrough et al., 2015). Such studies have extensively focused on live imaging of retinal blood flow dynamics of AD patients, yet there is a significant lack of understanding regarding the precise molecular and cellular mediators involved in retinal vascular AD pathology, which could lead to the discovery of potential intervention points of treatments and guide the development of next-generation retinal imaging. Thus, more in-depth investigations of the pathogenic mechanisms of retinal vasculature in AD development are needed.

To this end, work by Koronyo-Hamaoui and colleagues demonstrated the existence of different alloforms of Aβ deposits in retinal vessels of MCI and AD patients in both perivascular and within vessel walls including within the tunica media and outside the basement membrane (Koronyo et al., 2017). These findings were consistent with what is known regarding cerebral vascular Aβ pathology (Vinters and Gilbert, 1983; Vinters, 1987; Bennett, 2001; Bakker et al., 2016; Engelhardt et al., 2016). TEM analysis confirmed the ultrastructures of retinal Aβ deposits, often in close proximity to or within blood vessels, similar to those found in the brain (Iadanza et al., 2016; Koronyo et al., 2017; Shi et al., 2020). Retinal Aβ_42_ fibrils near a blood basal membrane were approximately 10-15nm in width with typical anti-parallel β-sheets (Koronyo et al., 2017). In the same human study, the existence of retinal Aβ was verified by Congo red staining under polarized light, by which the investigators revealed positive Aβ fibrils along blood vessels (Koronyo et al., 2017). According to an animal study with APP-overexpressing mice, vascular amyloidosis resulted in increased rigidity of the blood vessels and decreased blood flow in the brain (Kimbrough et al., 2015). In addition, a recent clinical study has proposed that cerebral vascular changes may propel other abnormalities in AD pathogenesis (Besser et al., 2016).

Recent reports suggest that vascular pathology in AD brains occurs very early during disease progression (Nikolakopoulou et al., 2017). Yet, it is unclear how early vascular pathology can be detected via retinal imaging and whether it may specifically foretell AD development. Furthermore, the connections between vascular pathology and Aβ accumulation and clearance in both the brain and retina should be explored in future studies. To date, several studies imply the potential of utilizing retinal vascular biomarkers for early AD or pre-symptomatic stage screening as well as for predicting cognitive decline (Vidal and Mavet, 1989; Gharbiya et al., 2014; Bulut et al., 2016; Cunha et al., 2017; McGrory et al., 2017; Bulut et al., 2018; Jiang et al., 2018; O’Bryhim et al., 2018; Jung et al., 2019).

**FIGURE 6:**
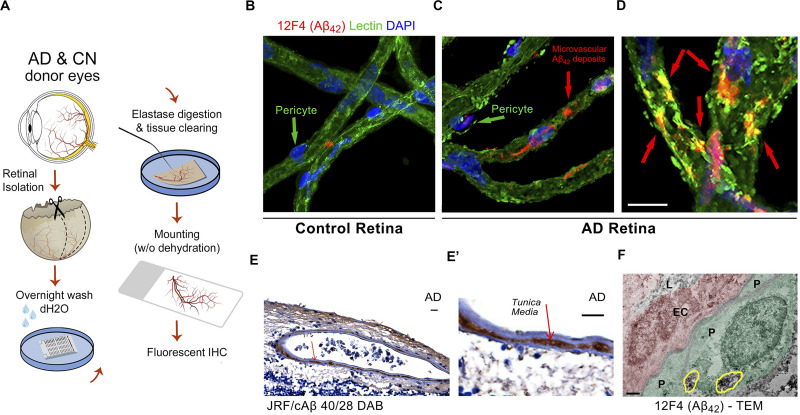
Identification of early and progressive PDGFRβ/pericyte loss, associated with vascular amyloidosis in AD retina. **(A)** Schematic diagram of retinal vascular network isolation and immunofluorescence staining. **(B,C)** Representative fluorescent images showing Aβ_42_ (12F4 immunoreactivity in red), blood vessels (lectin in green) and nuclei (DAPI in blue) in isolated retinal microvasculature networks from a cognitively normal (CN) control subject **(B)** and AD patient **(C)**, with higher Aβ_42_ deposits in AD retinal microvasculature and pericytes. **(D)** Higher magnification image of AD retina shows co-localization of Aβ_42_ and lectin-positive vascular walls in yellow. **(E,E’)** Retinal cross-section from an AD patient immunostained with anti-Aβ_40_ (JRF/cAβ_*40/28*_) mAb and DAB labeling and hematoxylin counterstain. Red arrow, also shown in higher magnification image **(E’)**, points to vascular Aβ_40_ in tunica media, adventitia or intima. Scale bar: 20 μm. **(F)** TEM image of retinal cross-section from an AD patient immunostained with anti-Aβ_42_ mAb (12F4) and peroxidase-based DAB, revealing the localization and ultrastructure of vascular-associated deposits. Cytoplasmic Aβ_42_ deposits in pericytes are demarcated by yellow lines. Scale bar: 0.5 μm. Reproduced from Shi et al. (2020) under terms of the Creative Commons Attribution 4.0 International License (http://creativecommons.org/licenses/by/4.0/).

A recent investigation by Shi and colleagues of retinal vasculature in 62 AD and MCI patients and matched human controls revealed early and progressive PDGFRβ deficiency and pericyte loss along with intense retinal vascular Aβ accumulation in AD (Shi et al., 2020). Using a modified retinal vascular isolation technique (Figure 6A) in 12 AD patient and control donors, the authors report a significant increase in various types of Aβ in AD retinal microvasculature, and more importantly, accumulation of Aβ in retinal pericytes together with pericyte loss (Figure 6B–E’). The existence of Aβ in pericytes was validated by TEM, which showed intense Aβ_42_ deposition in retinal pericytes as well as in microvascular lumen and adjacent to microvasculature (Figure 6F). Further, accumulation of Aβ in arterial tunica media in the retina of several AD patients (Figures 6E,E’; Shi et al., 2020) has implications related to lymphatic Aβ clearance pathways in the human retina: a subject warranting future exploration.

In the same study, histological analysis based on immunofluorescent staining or 3,3′-Diaminobenzidine staining and the use of specific monoclonal antibodies recognizing diverse Aβ epitopes revealed intense deposition of both Aβ_42_ and Aβ_40_ in AD retinal vasculature (*n* = 28–36 human samples). In addition, early loss of pericyte marker PDGFRβ was noted in longitudinal and vertical retinal vessels of MCI and AD patients compared to normal controls (*n* = 38). The dramatic loss of vascular PDGFRβ expression significantly correlated with CAA (*n* = 14) and Mini-Mental State Examination (MMSE) cognitive scores (*n* = 10) in a subset of patients, suggesting that retinal pericyte and PDGFRβ loss could predict the cerebral vascular disease and cognitive function. Further TUNEL and cleaved caspase-3 staining demonstrated that apoptosis may be the dominant pathway of retinal pericyte death in MCI and AD retina.

In the brain, the BBB plays an indispensable role in mediating clearance of Aβ through its efflux in the cerebral vascular network (Zlokovic et al., 1993; DeMattos et al., 2002; Banks et al., 2003; Do et al., 2015; Zhao et al., 2015; Sweeney et al., 2018). The BBB is established by endothelial cells forming vessel walls, astrocyte end-feet, and pericytes in the basement membrane. In comparison, the BRB is composed of tight junctions of retinal endothelial and epithelial cells together with supporting pericytes. Despite their organ-specific functions, the BBB and BRB display very similar functions in transport and permeation characteristics (Steuer et al., 2005). A recent study has established a correlation among BBB, ApoE ε4 and cognitive decline regardless of AD pathology (Montagne et al., 2020). The recent findings implicated pericytes in AD pathogenesis and future investigations may shed light on the role of the BRB in AD.

Permeability of the BRB may be measured in live laboratory animal models of retinal diseases by the extent of leakage using fluorescent dyes with predefined molecular weight such as fluorescein (Do carmo et al., 1998), Evans blue (Xu et al., 2001) and others (Ivanova et al., 2019). In the clinical setting, fundus fluorescein angiography (FFA) utilizing a fluorescent dye and fundus camera has been an established method for examining retinal vascular circulation and BRB damage (Marmor and Ravin, 2011). Recently, a newly modified OCT method was developed, the OCT-Leakage. By using a proprietary algorithm to identify sites of decreased optical reflectivity, the system quantifies and detects the correlation of retinal extracellular space with degrees of retinal edema (Cunha-Vaz et al., 2016; Cunha-Vaz, 2017). A live imaging study in 28 patients has demonstrated agreement between OCT-Leakage and FFA in identifying sites of impaired BRB in diabetes (Cunha-Vaz et al., 2017), providing a new noninvasive low-cost alternative method to detect and quantify BRB leakage.

Given that retinal vascular amyloidosis has been detected in AD (Koronyo et al., 2017; Shi et al., 2020), future studies should aim to examine if BRB permeability is also altered by disease, and if this is a cause or effect of retinal vascular amyloidosis. Indeed, a study using OCT angiography in patients revealed increased fovea avascular zone and decreased foveal thickness in eyes of AD patients (O’Bryhim et al., 2018), implying extensive retinal microvascular damage in the AD retina. Accordingly, recent human studies have demonstrated that retinal vascular abnormalities can predict cognitive decline (Baker et al., 2007; Cabrera DeBuc et al., 2018; Deal et al., 2018). Recent progress in retinal amyloid imaging (Koronyo et al., 2017), pericyte imaging by adaptive optics (Schallek et al., 2013) together with FFA and the recently developed OCT-Leakage (Cunha-Vaz et al., 2016) should allow for a comprehensive assessment of retinal Aβ pathology and BRB damage, potentially revolutionizing AD screening techniques.

## Retinal Inflammation in Alzheimer’s Disease

Chronic, low-grade inflammation is a typical sign of Alzheimer’s neuropathology (Akiyama et al., 2000). Neuroinflammation in AD is linked with increased astrocyte and microglia reactivity and neurotoxicity, Aβ and tau seeding and propagation, as well as with microglia-mediated synaptic pruning (Cardona et al., 2006; Wyss-Coray, 2006; Stevens et al., 2007; Fuhrmann et al., 2010; Lee et al., 2010; Wyss-Coray and Rogers, 2012; Hong et al., 2016; Salter and Stevens, 2017; Ising et al., 2019; Friker et al., 2020). As in the brain, some histological studies have implicated inflammation in the retina of AD patients (Liew et al., 1994; Blanks et al., 1996a; Grimaldi et al., 2018). This is particularly interesting as the eye has historically been considered an immune privileged site (Zhou and Caspi, 2010). In 1996, Blanks and colleagues discovered increased GFAP expression in retinal astrocytes and Müller cells in the GCL of AD retina, suggesting astrogliosis occurs in the retina of these patients (Blanks et al., 1996a). More recently, detrimental astrocyte and microglial activation was observed along with Aβ plaques, tau tangles, and neurodegeneration in postmortem retinal tissues of 6 AD patients as compared with 6 control subjects (Grimaldi et al., 2019). Notably, molecular mediators of innate immunity including interleukin-1β (IL-1β), complement component 3 (C3), osteopontin, and triggering receptor expressed on myeloid cells 2 were found to be upregulated in retinal tissues of AD patients (Grimaldi et al., 2019).

Although much work in larger retinal samples is needed to investigate the nature and potential mechanisms of retinal inflammation in human AD, various studies in animal models of AD have also provided evidence and insights into retinal inflammation in this disorder. Ning and colleagues first established the correlation of neurodegeneration and inflammation in the retina of ADtg mice (Ning et al., 2008). Other studies have shown increased microgliosis and astrocytosis, infiltration of lymphocytes and monocytes, and upregulation of monocyte chemoattractant protein-1 (MCP-1) in multiple layers of the retina and choroid (Ning et al., 2008; Liu et al., 2009; Perez et al., 2009; Yang et al., 2013; Edwards et al., 2014; Tsai et al., 2014; Antes et al., 2015; Gao et al., 2015; Pogue et al., 2015). A recent study in 3xTg AD mice has further described morphological changes in retinal microglia, including increased microglial cell number, soma size, retraction and reorientation of microglial processes, and change in cell locations (Salobrar-Garcia et al., 2020).

In general, retinal inflammation is implicated in multiple traditional retinal vascular and neurodegenerative disorders, including diabetic retinopathy (Tang and Kern, 2011; Semeraro et al., 2015; Rubsam et al., 2018), AMD (Knickelbein et al., 2015; Kauppinen et al., 2016), and glaucoma (Vohra et al., 2013). During the onset of disease pathogenesis, retinal inflammation is usually triggered by an imbalance of pro- versus anti-inflammatory molecules. This can be evoked by a wide spectrum of pathogenic pathways, including overproduction of reactive oxygen species, activation of NF-κB or protein kinase C pathways, inflammasome or microglial activation, advanced glycation end products, or shear pressure and leukocyte invasion due to retinal microvascular damage. In this context, the recent discovery of early retinal pericyte loss in MCI patients (Shi et al., 2020) suggests an early BRB disturbance and perhaps retinal microvascular leakage in AD pathogenesis that may be implicated in retinal inflammation. Future studies should evaluate BRB leakage and the potential relationship with imbalanced retinal inflammatory pathway activation and brain inflammation in AD. Findings from such studies could lead to the discovery of novel retinal biomarkers to facilitate AD detection and monitoring.

## Functional and Visual Changes in Alzheimer’s Disease

As discussed above, the retina undergoes neuropathological changes similar to the brain in AD patients. This is not surprising given the developmental, physiological, and anatomical similarities between the two tissue types, rendering them vulnerable to AD-related neuronal and functional abnormalities (Byerly and Blackshaw, 2009; London et al., 2013; Jindal, 2015). A host of visual and ocular manifestations were reported in both MCI and AD patients, particularly loss of contrast sensitivity, color discrimination deficits, difficulties with depth and motion perception, circadian rhythm irregularities, and sleep disturbances (Sadun et al., 1987; Katz and Rimmer, 1989; Trick et al., 1989; Cronin-Golomb et al., 1991; Cronin-Golomb, 1995; Lakshminarayanan et al., 1996; Cormack et al., 2000; Rizzo et al., 2000; Pache et al., 2003; Ng et al., 2007; Armstrong, 2009; Salamone et al., 2009; Chang et al., 2014; Nolan et al., 2014; Armstrong and Kergoat, 2015; Salobrar-Garcia et al., 2015; Hart et al., 2016; Javaid et al., 2016; La Morgia et al., 2016; Polo et al., 2017; Cerquera-Jaramillo et al., 2018; Colligris et al., 2018; Risacher et al., 2020). Importantly, retinal changes in AD patients such as RGC degeneration, reduced NFL thickness, and decreased blood circulation were linked to specific visual and ocular disturbances (Hinton et al., 1986; Blanks et al., 1989, 1996a, b; Parisi et al., 2001; Berisha et al., 2007; Paquet et al., 2007; Kesler et al., 2011).

Alzheimer’s hallmark pathologies Aβ deposits and tauopathy are found in retinal cell types and topographical regions shown to undergo degeneration and physiological abnormalities in both patients and transgenic animal models (Koronyo-Hamaoui et al., 2011; Schön et al., 2012; La Morgia et al., 2016; Koronyo et al., 2017; den Haan et al., 2018; Asanad et al., 2019b; Grimaldi et al., 2019; Habiba et al., 2020; Shi et al., 2020). These findings provide a link between AD-related retinal neuropathology and the visual deficits reported in living patients. For instance, histological examination of retinal tissue shows colocalization of pTau and Aβ pathology in regions associated with RGC loss (Schön et al., 2012; La Morgia et al., 2016; Koronyo et al., 2017; den Haan et al., 2018; Asanad et al., 2019b; Grimaldi et al., 2019; Shi et al., 2020). Specifically, degeneration of RGCs would undoubtedly compromise their essential role in receiving sensory input from photoreceptors via bipolar cells, and in early stages of visual information processing and conveyance to the brain (Demb and Singer, 2015). In support of this, a recent study utilizing OCT and pattern electroretinography (ERG) in AD patients showed a linear relationship between NFL thickness and the bioelectrical integrity of RGCs and their nerve fibers, which are essential for afferent signal transduction (Ngoo et al., 2019).

In addition to the above reported changes, irregularities in efferent ocular pathways in conjunction with retinal abnormalities were also associated with altered pupillary light response (PLR) in patients. These alterations include increased latency of pupillary constriction to light, decreased constriction amplitude, faster redilation after light offset, decreased maximum constriction velocity and acceleration (Chougule et al., 2019), and altered pupil dilation response during cognitive tasks (Granholm et al., 2017; Kremen et al., 2019). Therefore, there is growing interest in exploring various aspects of pupillary responses, using specialized pupillometry tools, in pre-clinical and clinically diagnosed AD patients. Further to being governed by both the sympathetic and parasympathetic systems, PLR is also regulated by retinal rods, cones, and mRGCs (Hattar et al., 2003). In line with this, altered PLR in AD patients was associated with retinal mRGC loss (La Morgia et al., 2016), highlighting the consequences of retinal AD pathology for various ocular and visual dysfunctions. Moreover, these intrinsically photosensitive RGCs are also reported to regulate light photoentrainment of circadian rhythms, supported by their tightly regulated communication with photoreceptors and their projections to the central circadian clock in the hypothalamic suprachiasmatic nucleus (SCN) (Lu et al., 1999; Berson et al., 2002; Gooley et al., 2003; Markwell et al., 2010; Chen et al., 2011). Therefore, retinal mRGC loss is suggested to play a key role in the sleep-wake cycle dysfunctions observed in AD patients (La Morgia et al., 2016, 2017). Sleep abnormalities reported in MCI and AD patients include prolonged sleep latency, reduced total sleep duration and rapid eye movement (REM) sleep, sleep fragmentation, frequent awakenings and lower melatonin levels at night, and daytime somnolence (Petit et al., 2004; Ju et al., 2013; Peter-Derex et al., 2015; Feng et al., 2016; La Morgia et al., 2016, 2017; Weissová et al., 2016; Asanad et al., 2019b). Intriguingly, there are reports of sleep disturbances exacerbating or even preceding cognitive impairments (Ju et al., 2014; Bubu et al., 2017; Brzecka et al., 2018). The essential discovery that Aβ accumulates within or in close proximity to mRGCs, which undergo degeneration and dendrite diameter loss, at least in part, can explain impaired sleep patterns and altered pupil dilation in AD patients (La Morgia et al., 2016). These findings also suggest that certain retinal cells are more susceptible to Aβ-induced neurotoxicity and proposes the first retinal damage-based mechanism for functional disturbances commonly seen in AD patients.

The spatial distribution of retinal AD pathology may indicate the specific functional deficit. Regionally, both Aβ and pTau pathologies appear to preferentially affect the peripheral superior and inferior retinal quadrants in AD patients (Koronyo-Hamaoui et al., 2011; La Morgia et al., 2016; Koronyo et al., 2017; den Haan et al., 2018). This, along with consistent neuronal loss, degeneration of mRGCs and RGCs, and NFL thinning in these particular peripheral regions, could underlie visual abnormalities such as in contrast sensitivity, motion perception and circadian rhythms described in AD (Trick et al., 1989, 1995; Blanks et al., 1996a; Berisha et al., 2007; Lu et al., 2010; Kesler et al., 2011; Moschos et al., 2012; Ju et al., 2013; Kirbas et al., 2013; Risacher et al., 2013; Kromer et al., 2014; La Morgia et al., 2016). Beyond AD-related manifestations predominantly in the inner layers and peripheral retinal regions, deposits of Aβ were found in close proximity to blood vessels and within vessel walls (Koronyo-Hamaoui et al., 2011; La Morgia et al., 2016; Koronyo et al., 2017; Shi et al., 2020). Furthermore, vascular amyloidosis positively correlated with the degree of vascular PDGFR-β deficiency as well as Aβ_40_ and Aβ_42_ alloforms accumulated within degenerating pericytes in MCI and AD retinas (Koronyo et al., 2017; Shi et al., 2020). These findings, together with altered retinal neurovascular coupling and structural vascular abnormalities in clinically diagnosed MCI and AD patients (Berisha et al., 2007; Querques et al., 2019), implicate retinal vascular-associated Aβ in causing or amplifying retinal metabolic dysfunctions and impaired clearance pathways. These changes may lead to functional-visual abnormalities.

A study in monkeys has recently provided evidence for the involvement of mRGCs in blue color sensitivity (Dacey et al., 2005). AD patients are reported to experience altered color vision with a selective and severity-independent deficiency in blue hue discrimination (Cronin-Golomb et al., 1993; Wijk et al., 1999; Rizzo et al., 2000; Chang et al., 2014; Polo et al., 2017). Interestingly, impaired blue color discrimination also manifests in patients with other neurodegenerative disorders (Paulus et al., 1993; Haug et al., 1995; Birch et al., 1998; Rodnitzky, 1998; Melun et al., 2001; Anssari et al., 2020).

The second most common cause of irreversible vision loss is glaucoma, a condition characterized by optic neuropathy, apoptotic RGC degeneration and NFL damage, driven by increased IOP and accumulation of proteinaceous deposits of Aβ and pTau (Cordeiro et al., 2004). Indeed, a higher occurrence of glaucoma was reported in AD patients compared to controls (Bayer et al., 2002; Tamura et al., 2006). Diminished contrast sensitivity, altered motion and depth perception, and oculomotor disturbances in both AD and glaucoma patients are partially attributed to a preferential loss of the functionally relevant magnocellular RGCs (M-cells) and their nerve fibers (Sadun and Bassi, 1990; Fiorentini et al., 1996; La Morgia et al., 2017). Moreover, impaired contrast sensitivity in MCI patients was shown to significantly correlate with the cerebral burden of amyloid and tau pathology (Risacher et al., 2020). Given the higher concentration of rods in the peripheral retina and their importance in motion and depth perception, low-contrast sensitivity, and peripheral vision, it is not surprising that these visual functions may be adversely influenced by the increased levels of Aβ deposits and pTau in the periphery (Gilmore et al., 1994; Risacher et al., 2013). Cumulative evidence of vision loss and ocular impairments accompanied by progressive retinal changes in AD and other neurodegenerative disorders, including glaucoma, multiple sclerosis (MS), PD, and Huntington’s disease (HD), robustly support the involvement of specific retinal pathologies in functional and visual manifestations of these conditions (Fisher et al., 2006; Gupta et al., 2008; Archibald et al., 2009; Green et al., 2010; London et al., 2013; Kersten et al., 2015; Andrade et al., 2016; Weil et al., 2016; La Morgia et al., 2017).

Despite many recent advancements, the field of AD retinopathy is still in its infancy and more studies focusing on mechanistic and causative factors are necessary to determine the connection between specific retinal pathologies, neuronal-type impairments and visual-functional deficits.

## Retinal Imaging

The mounting case for AD pathology in the retina has motivated investigations to develop various retinal imaging modalities, beginning with blue-light high-resolution photography by Tsai et al., 1991, which indicated a correlation between Alzheimer’s Disease Assessment Scale (ADAS) scores and optic nerve head changes in AD patients (Tsai et al., 1991).

Further studies employed cross-sectional imaging by optical coherence tomography (OCT) or optical coherence tomography angiography (OCTA) (Parisi et al., 2001; Kromer et al., 2014; Cunha et al., 2016; Ferrari et al., 2017; Polans et al., 2017; Polo et al., 2017; Bulut et al., 2018; Janez-Escalada et al., 2019; Salobrar-Garcia et al., 2019), which revealed structural abnormalities and cell neurodegeneration in the retina.

**FIGURE 7:**
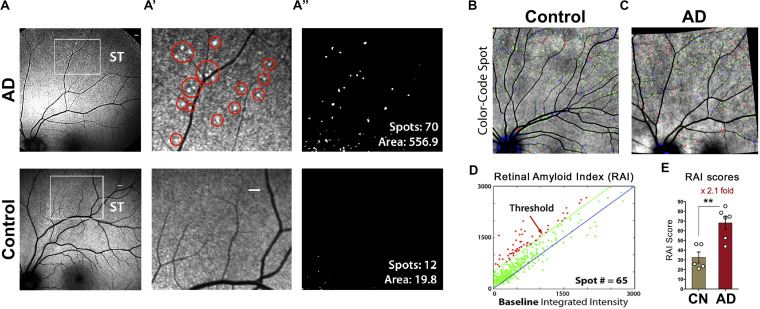
Proof-of-concept clinical trial shows the feasibility of noninvasive *in vivo* retinal curcumin-amyloid deposit imaging in AD patients. **(A)** Representative images of curcumin fluorescence fundography, enabling detection of increased retinal curcumin spots in a living AD patient relative to minimal spots in a cognitively normal (CN) control subject; Regions of interest (ROI) in superotemporal (ST) retinas are demarcated by white rectangles. Scale bar: 400 μm. **(A’)** Higher magnification image of the ROI with red circles highlighting curcumin-amyloid deposits in peripheral region of AD retina. **(A”)** Representative postprocessing images used to quantify spot number and fluorescent area (μm^2^). **(B,C)** color-coded overlay images from CN **(B)** and AD **(C)** retinas with curcumin-positive amyloid deposits above threshold shown in red, spots above 1:1 reference but below threshold shown in green and spots below reference in blue. **(D)** Representative graph showing number of color-coded spots (described in **B,C**) in AD retina used to determine retinal amyloid index (RAI) score. **(E)** Comparison of RAI scores in AD patients (*n* = 6) and age-matched CN subjects (*n* = 5). Data shown as group mean **±** SEM. **P < 0.005, unpaired 2-tailed Student’s *t*-test. Reproduced from Koronyo et al. (2017) with permission of ASCI via Copyright Clearance Center.

As mentioned above, the first *in vivo* imaging of AD hallmark pathology was initially developed and tested in transgenic murine models of AD by Koronyo-Hamaoui and colleagues (Koronyo-Hamaoui et al., 2011; Koronyo et al., 2012). The researchers paired curcumin – a natural and safe fluorochrome that labels Aβ fibrils and oligomers with high affinity and specificity – with a modified rodent retinal optical imaging microscope. Later, this approach was translated to humans and the feasibility to noninvasively detect and quantify individual retinal amyloid plaques was demonstrated in living patients with a modified confocal scanning laser ophthalmoscope following oral administration of highly bioavailable Longvida curcumin (Figures 7A–D; Garcia-Alloza et al., 2007; Gota et al., 2010; Koronyo-Hamaoui et al., 2011; Koronyo et al., 2012, 2017). Studies in murine ADtg models demonstrated the feasibility to longitudinally monitor individual Aβ deposits including their appearance and clearance during disease progression and in response to immune-based therapy (Koronyo et al., 2012).

Importantly, in the proof-of-principal clinical trial, this noninvasive amyloid imaging technique revealed a significant 2.1-fold greater retinal amyloid burden, termed as retinal amyloid index (RAI), in a group of 10 AD patients as compared to 6 age-matched healthy controls (Figure 7E; Koronyo et al., 2017). Supported by histological findings, retinal Aβ deposits were often found the mid- and far-periphery of the superior and inferior regions, where previously NFL thinning was reported as more pronounced. Further, amyloid deposits were found above the retinal pigment epithelium in the neurosensory retina, unlike the typical location of drusen (Koronyo et al., 2017). Anecdotal data from 2 AMD patients suggested that retinal curcumin fluorescence signals were diffuse and concentrated at the posterior pole, apparently distinct from findings in the retinas of AD patients (Koronyo et al., 2017). Moreover, results presented at the Alzheimer’s Association International Conference on July 15, 2014, from a large cohort of over 150 MCI, AD, and cognitively normal participants of the Australian Imaging, Biomarker and Lifestyle (AIBL) Study (www.aibl.csiro.au) found that retinal amyloid fluorescence imaging predicted cerebral amyloid burden and was significantly higher in AD patients (Frost et al., 2014).

Expanding on this work, Kayabasi and colleagues detected abnormal Aβ deposits in 30 MCI patients using fundus autofluorescence imaging (FAF) and OCT (Kayabasi et al., 2014). An additional study utilizing OCT revealed a correlation between retinal inclusion bodies and cortical amyloid burden in pre-clinical patients via florbetapir PET and multiple retinal sd-OCT aggregation markers of possible disease burden (Snyder et al., 2016).

Noninvasive *in vivo* retinal hyperspectral imaging (rHSI) has also recently been employed in patients and controls. One study found significant differences in the retinal reflectance spectra of MCI patients with high Aβ burden, confirmed by brain PET imaging, as compared to age-matched controls, validated in an independent cohort with a second hyperspectral camera (Hadoux et al., 2019). Retinal imaging scores correlated with Aβ burden in the brain (Hadoux et al., 2019). Intriguingly, a similar study from More and colleagues, the team that initially developed the rHSI technique in murine models (More and Vince, 2015; More et al., 2016), found the largest deviation in rHSI signatures between MCI patients and controls, irrespective of other ocular conditions such as cataracts or glaucoma (More et al., 2019). The same technique was able to distinguish cerebral Aβ+ subjects from Aβ− subjects with 85% accuracy based on retinal vascular measures including vessel tortuosity and diameter (Sharafi et al., 2019).

Finally, a recent multimodal approach including visual performance tests, advanced retinal imaging, and full-field electroretinogram (ERG) in 69 cognitively impaired subjects revealed sensitivity of the combined approach to cognitive decline (Cabrera DeBuc et al., 2018). Although more robust investigations are needed to validate these findings, this study supports the association of retinal geometric vascular and functional parameters with physiological changes in the retina in cognitively impaired individuals (Cabrera DeBuc et al., 2018). Future tools should assess whether a combination of these multimodal methods with specific retinal amyloid imaging will allow for earlier and/or more accurate assessment of AD.

## Conclusion

With the number of AD patients estimated to triple by 2050 (Alzheimer’s Association, 2019), a reliable, noninvasive and affordable diagnostic technique suitable for widespread clinical use is urgently needed. In recent years, a radical idea has emerged that AD pathology in the brain can also manifest and perhaps be mirrored in the retina. Indeed, the identification of hallmark Aβ deposits in the retina of patients and numerous animal models and their correlation with pathology in the brain have fueled this field of neuro-ophthalmology in AD. Moreover, mounting evidence now supports the existence of diffuse, classical and neuritic-like plaques, pTau aggregates, Aβ_42_ fibrils, protofibrils, and oligomer-like structures, pericyte loss, vascular Aβ_40_ and Aβ_42_ accumulation, inflammation and neurodegeneration in the retina of AD patients (Alexandrov et al., 2011; Koronyo-Hamaoui et al., 2011; Schön et al., 2012; Tsai et al., 2014; La Morgia et al., 2016; Koronyo et al., 2017; den Haan et al., 2018; Grimaldi et al., 2018; Hadoux et al., 2019; Schultz et al., 2020; Shi et al., 2020). Despite many recent successes, several limitations including the scarcity of retinal tissue from people with neuropathological data and the application of non-conventional histological and biochemical techniques to examine AD effects in various topographical regions, has historically restricted the knowledge in this field. Future investigations, involving brain biobanks with extended collection of ocular tissues, will allow researchers to determine how early AD pathological processes occur in the retina, their extent, distribution, and relationship to brain pathology.

Histological and biochemical examinations of postmortem retina from AD patients and animal models have proven invaluable in deciphering the pathological burden of disease hallmarks, susceptible retinal layers and cell types, and putative molecular pathways linked to retinal atrophy and functional deficits. Nevertheless, there is still lack of understanding of the impact of Aβ and tau pathologies on retinal cell types; some effects, including nerve fiber thinning, neuronal loss and vascular changes, may not manifest in early stages of disease and may not be specific to AD. For instance, reduced NFL, macular, and foveal thickness have also been observed via OCT examinations of Parkinson’s disease (PD) patient retinas as compared to normal controls (Inzelberg et al., 2004; Altintas et al., 2008; Hajee et al., 2009; Cubo et al., 2010; Moschos et al., 2011, 2012; Shrier et al., 2012; Adam et al., 2013; Moreno-Ramos et al., 2013; Satue et al., 2013; Moschos and Chatziralli, 2017).

Notably, according to the 2018 NIA-AA research framework, the presence of Aβ deposits is a requirement for Alzheimer’s pathological change diagnosis and should be considered in early screening efforts and recruitment of individuals for clinical trials (Jack et al., 2018). The ability to detect and quantify Aβ deposits and related pathologies via noninvasive retinal imaging along with sensitive, routine, minimally invasive plasma AD biomarkers show great promise for diagnostic screening, monitoring of progression, and assessment of therapeutic efficacy (Hampel et al., 2018; Baldacci et al., 2020). The work described here combined with future advances may lead to clinical translation, discrimination of pathophysiological phenotypes during the AD continuum, and eventually a highly anticipated cure for this destructive disease.

Future investigations should focus on standardization of histological techniques, identification of early AD biomarkers in the retina, their spatiotemporal profiles, and enhancing the sensitivity of retinal imaging modalities for detection of these pathological indicators in this new and expanding field of Alzheimer’s retinopathy.

## Author Contributions

NM, HS, MO, and MK-H drafted manuscript. NM, HS, JD, YK, and MK-H did the figure and image preparation. NM, HS, MO, JD, AR, D-TF, JS, KB, YK, and MK-H edited the manuscript. MK-H did the study supervision. All authors read and approved the final manuscript.

## Conflict of Interest

YK, MK-H, and KB were co-founders and stockholders of NeuroVision Imaging, Inc., 1395 Garden Highway, Suite 250, Sacramento, CA, United States. The remaining authors declare that the research was conducted in the absence of any commercial or financial relationships that could be construed as a potential conflict of interest.

## References

[B1] Abbasi J. (2017). A retinal scan for Alzheimer disease. *JAMA* 318 1314. 10.1001/jama.2017.15192 29049570

[B2] Aboelnour A. Van Der Spuy J. Powner M. Jeffery G. (2017). Primate retinal cones express phosphorylated tau associated with neuronal degeneration yet survive in old age. *Exp. Eye Res.* 165 105–108. 10.1016/j.exer.2017.09.013 28974357 PMC5725308

[B3] Adam C. R. Shrier E. Ding Y. Glazman S. Bodis-Wollner I. (2013). Correlation of inner retinal thickness evaluated by spectral-domain optical coherence tomography and contrast sensitivity in Parkinson disease. *J. Neuroophthalmol.* 33 137–142. 10.1097/WNO.0b013e31828c4e1a 23612240

[B4] Akiyama H. Barger S. Barnum S. Bradt B. Bauer J. Cole G. M. (2000). Inflammation and Alzheimer’s disease. *Neurobiol. Aging* 21 383–421. 10.1016/S0197-4580(00)00124-X 10858586 PMC3887148

[B5] Alexandrov P. N. Pogue A. Bhattacharjee S. Lukiw W. J. (2011). Retinal amyloid peptides and complement factor H in transgenic models of Alzheimer’s disease. *Neuroreport* 22 623–627. 10.1097/WNR.0b013e3283497334 21734608 PMC3719862

[B6] Allen B. Ingram E. Takao M. Smith M. J. Jakes R. Virdee K. (2002). Abundant tau filaments and nonapoptotic neurodegeneration in transgenic mice expressing human P301S tau protein. *J. Neurosci.* 22 9340–9351. 10.1523/JNEUROSCI.22-21-09340.2002 12417659 PMC6758022

[B7] Altintas O. Iseri P. Ozkan B. Caglar Y. (2008). Correlation between retinal morphological and functional findings and clinical severity in Parkinson’s disease. *Doc. Ophthalmol.* 116 137–146. 10.1007/s10633-007-9091-8 17962989

[B8] AlzBiomarker Database (2018). *AlzBiomaker Database, Version 2.0 [Online].* Available: https://www.alzforum.org/alzbiomarker (accessed on 2020).

[B9] Alzheimer’s Association (2019). 2019 Alzheimer’s disease facts and figures. *Alzheimer’s Dement.* 15 321–387. 10.1016/j.jalz.2019.01.010

[B10] Anderson D. H. Talaga K. C. Rivest A. J. Barron E. Hageman G. S. Johnson L. V. (2004). Characterization of beta amyloid assemblies in drusen: the deposits associated with aging and age-related macular degeneration. *Exp. Eye Res.* 78 243–256. 10.1016/j.exer.2003.10.011 14729357

[B11] Andrade C. Beato J. Monteiro A. Costa A. Penas S. Guimarães J. (2016). Spectral-domain optical coherence tomography as a potential biomarker in Huntington’s Disease. *Mov. Disord.* 31 377–383. 10.1002/mds.26486 26853218

[B12] Anssari N. Vosoughi R. Mullen K. Mansouri B. (2020). Selective colour vision deficits in multiple sclerosis at different temporal stages. *Neuroophthalmology* 44 16–23. 10.1080/01658107.2019.1615960 32076444 PMC6999627

[B13] Antes R. Salomon-Zimri S. Beck S. C. Garcia Garrido M. Livnat T. Maharshak I. (2015). VEGF mediates ApoE4-induced neovascularization and synaptic pathology in the Choroid and Retina. *Curr. Alzheimer Res.* 12 323–334. 10.2174/1567205012666150325182504 25817253

[B14] Archibald N. K. Clarke M. P. Mosimann U. P. Burn D. J. (2009). The retina in Parkinson’s disease. *Brain* 132 1128–1145. 10.1093/brain/awp068 19336464

[B15] Ardiles A. O. Tapia-Rojas C. C. Mandal M. Alexandre F. Kirkwood A. Inestrosa N. C. (2012). Postsynaptic dysfunction is associated with spatial and object recognition memory loss in a natural model of Alzheimer’s disease. *Proc. Natl. Acad. Sci. U.S.A.* 109 13835–13840. 10.1073/pnas.1201209109 22869717 PMC3427050

[B16] Armstrong R. Kergoat H. (2015). Oculo-visual changes and clinical considerations affecting older patients with dementia. *Ophthal. Physiol. Opt.* 35 352–376. 10.1111/opo.12220 26094831

[B17] Armstrong R. A. (2009). Alzheimer’s disease and the eye. *J. Opt.* 2 103–111. 10.3921/joptom.2009.103

[B18] Arvanitakis Z. Leurgans S. E. Wang Z. Wilson R. S. Bennett D. A. Schneider J. A. (2011). Cerebral amyloid angiopathy pathology and cognitive domains in older persons. *Ann. Neurol.* 69 320–327. 10.1002/ana.22112 21387377 PMC3228518

[B19] Asanad S. Ross-Cisneros F. N. Barron E. Nassisi M. Sultan W. Karanjia R. (2019a). The retinal choroid as an oculovascular biomarker for Alzheimer’s dementia: a histopathological study in severe disease. *Alzheimers Dement.* 11 775–783. 10.1016/j.dadm.2019.08.005 31737776 PMC6849152

[B20] Asanad S. Ross-Cisneros F. N. Nassisi M. Barron E. Karanjia R. Sadun A. A. (2019b). The Retina in Alzheimer’s disease: histomorphometric analysis of an ophthalmologic biomarker. *Invest. Ophthalmol. Vis. Sci.* 60 1491–1500. 10.1167/iovs.18-25966 30973577 PMC6892387

[B21] Avila J. Pallas N. Bolos M. Sayas C. L. Hernandez F. (2016). Intracellular and extracellular microtubule associated protein tau as a therapeutic target in Alzheimer disease and other tauopathies. *Expert. Opin. Ther. Targets* 20 653–661. 10.1517/14728222.2016.1131269 26652296

[B22] Bakalash S. Pham M. Koronyo Y. Salumbides B. C. Kramerov A. Seidenberg H. (2011). Egr1 expression is induced following glatiramer acetate immunotherapy in rodent models of glaucoma and Alzheimer’s disease. *Invest. Ophthalmol. Vis. Sci.* 52 9033–9046. 10.1167/iovs.11-7498 21969301

[B23] Baker M. L. Marino Larsen E. K. Kuller L. H. Klein R. Klein B. E. Siscovick D. S. (2007). Retinal microvascular signs, cognitive function, and dementia in older persons: the Cardiovascular Health Study. *Stroke* 38 2041–2047. 10.1161/STROKEAHA.107.483586 17525385

[B24] Bakker E. N. Bacskai B. J. Arbel-Ornath M. Aldea R. Bedussi B. Morris A. W. (2016). Lymphatic clearance of the brain: perivascular, paravascular and significance for neurodegenerative diseases. *Cell Mol. Neurobiol.* 36 181–194. 10.1007/s10571-015-0273-8 26993512 PMC4844641

[B25] Baldacci F. Mazzucchi S. Della Vecchia A. Giampietri L. Giannini N. Koronyo-Hamaoui M. (2020). The path to biomarker-based diagnostic criteria for the spectrum of neurodegenerative diseases. *Expert Rev. Mol. Diagn.* 20 421–441. 10.1080/14737159.2020.1731306 32066283 PMC7445079

[B26] Banks W. A. Robinson S. M. Verma S. Morley J. E. (2003). Efflux of human and mouse amyloid beta proteins 1-40 and 1-42 from brain: impairment in a mouse model of Alzheimer’s disease. *Neuroscience* 121 487–492. 10.1016/S0306-4522(03)00474-3 14522007 PMC3389491

[B27] Baruch K. Deczkowska A. Rosenzweig N. Tsitsou-Kampeli A. Sharif A. M. Matcovitch-Natan O. (2016). PD-1 immune checkpoint blockade reduces pathology and improves memory in mouse models of Alzheimer’s disease. *Nat. Med.* 22 135–137. 10.1038/nm.4022 26779813

[B28] Bateman R. J. Xiong C. Benzinger T. L. Fagan A. M. Goate A. Fox N. C. (2012). Clinical and biomarker changes in dominantly inherited Alzheimer’s disease. *N. Engl. J. Med.* 367 795–804. 10.1056/NEJMoa1202753 22784036 PMC3474597

[B29] Bayer A. U. Ferrari F. Erb C. (2002). High occurrence rate of glaucoma among patients with Alzheimer’s disease. *Eur. Neurol.* 47 165–168. 10.1159/000047976 11914555

[B30] Behbehani R. Adnan H. Al-Hassan A. A. Al-Salahat A. Alroughani R. (2018). Predictors of retinal atrophy in multiple sclerosis: a longitudinal study using spectral domain optical coherence tomography with segmentation analysis. *Multiple Scler. Relat. Disord.* 21 56–62. 10.1016/j.msard.2018.02.010 29459346

[B31] Bennett D. (2001). Public health importance of vascular dementia and Alzheimer’s disease with cerebrovascular disease. *Int. J. Clin. Pract. Suppl.* 120, 41–48. 11406925

[B32] Berisha F. Feke G. T. Trempe C. L. Mcmeel J. W. Schepens C. L. (2007). Retinal abnormalities in early Alzheimer’s disease. *Invest. Ophthalmol. Vis. Sci.* 48 2285–2289. 10.1167/iovs.06-1029 17460292

[B33] Bernstein K. E. Koronyo Y. Salumbides B. C. Sheyn J. Pelissier L. Lopes D. H. (2014). Angiotensin-converting enzyme overexpression in myelomonocytes prevents Alzheimer’s-like cognitive decline. *J. Clin. Invest.* 124 1000–1012. 10.1172/JCI66541 24487585 PMC3934162

[B34] Berson D. M. Dunn F. A. Takao M. (2002). Phototransduction by retinal ganglion cells that set the circadian clock. *Science* 295 1070–1073. 10.1126/science.1067262 11834835

[B35] Besser L. M. Alosco M. L. Ramirez Gomez L. Zhou X. H. Mckee A. C. Stern R. A. (2016). Late-life vascular risk factors and Alzheimer disease neuropathology in individuals with normal cognition. *J. Neuropathol. Exp. Neurol.* 75 955–962. 10.1093/jnen/nlw072 27516116 PMC5029440

[B36] Bilgel M. An Y. Zhou Y. Wong D. F. Prince J. L. Ferrucci L. (2016). Individual estimates of age at detectable amyloid onset for risk factor assessment. *Alzheimers Dement.* 12 373–379. 10.1016/j.jalz.2015.08.166 26588863 PMC4841700

[B37] Birch J. Kolle R. U. Kunkel M. Paulus W. Upadhyay P. (1998). Acquired colour deficiency in patients with Parkinson’s disease. *Vision Res.* 38 3421–3426. 10.1016/S0042-6989(97)00398-2 9893859

[B38] Blanks J. C. Hinton D. R. Sadun A. A. Miller C. A. (1989). Retinal ganglion cell degeneration in Alzheimer’s disease. *Brain Res.* 501 364–372. 10.1016/0006-8993(89)90653-7 2819446

[B39] Blanks J. C. Schmidt S. Y. Torigoe Y. Porrello K. V. Hinton D. R. Blanks R. H. (1996a). Retinal pathology in Alzheimer’s disease. II. Regional neuron loss and glial changes in GCL. *Neurobiol. Aging* 17 385–395. 10.1016/0197-4580(96)00009-7 8725900

[B40] Blanks J. C. Torigoe Y. Hinton D. R. Blanks R. H. (1996b). Retinal pathology in Alzheimer’s disease. I. Ganglion cell loss in foveal/parafoveal retina. *Neurobiol. Aging* 17 377–384. 10.1016/0197-4580(96)00010-3 8725899

[B41] Bookheimer S. Y. Strojwas M. H. Cohen M. S. Saunders A. M. Pericak-Vance M. A. Mazziotta J. C. (2000). Patterns of brain activation in people at risk for Alzheimer’s disease. *N. Engl. J. Med.* 343 450–456. 10.1056/NEJM200008173430701 10944562 PMC2831477

[B42] Brzecka A. Leszek J. Ashraf G. M. Ejma M. Ávila-Rodriguez M. F. Yarla N. S. (2018). Sleep disorders associated with Alzheimer’s disease: a perspective. *Front. Neurosci.* 12:330. 10.3389/fnins.2018.00330 29904334 PMC5990625

[B43] Bubu O. M. Brannick M. Mortimer J. Umasabor-Bubu O. Sebastião Y. V. Wen Y. (2017). Sleep, cognitive impairment, and Alzheimer’s disease: a systematic review and meta-analysis. *Sleep* 40:zsw032. 10.1093/sleep/zsw032 28364458

[B44] Buckley R. F. Hanseeuw B. Schultz A. P. Vannini P. Aghjayan S. L. Properzi M. J. (2017). Region-specific association of subjective cognitive decline with tauopathy independent of global β-Amyloid Burden. *JAMA Neurol.* 74 1455–1463. 10.1001/jamaneurol.2017.2216 28973551 PMC5774633

[B45] Bulut M. Kurtulus F. Gozkaya O. Erol M. K. Cengiz A. Akidan M. (2018). Evaluation of optical coherence tomography angiographic findings in Alzheimer’s type dementia. *Br. J. Ophthalmol.* 102 233–237. 10.1136/bjophthalmol-2017-310476 28600299

[B46] Bulut M. Yaman A. Erol M. K. Kurtuluş F. Toslak D. Doğan B. (2016). Choroidal thickness in patients with mild cognitive impairment and Alzheimer’s type dementia. *J. Ophthalmol.* 2016:7291257. 10.1155/2016/7291257 26925259 PMC4748862

[B47] Butovsky O. Koronyo-Hamaoui M. Kunis G. Ophir E. Landa G. Cohen H. (2006). Glatiramer acetate fights against Alzheimer’s disease by inducing dendritic-like microglia expressing insulin-like growth factor 1. *Proc. Natl. Acad. Sci. U.S.A.* 103 11784–11789. 10.1073/pnas.0604681103 16864778 PMC1544247

[B48] Butovsky O. Kunis G. Koronyo-Hamaoui M. Schwartz M. (2007). Selective ablation of bone marrow-derived dendritic cells increases amyloid plaques in a mouse Alzheimer’s disease model. *Eur. J. Neurosci.* 26 413–416. 10.1111/j.1460-9568.2007.05652.x 17623022

[B49] Byerly M. S. Blackshaw S. (2009). Vertebrate retina and hypothalamus development. *Wiley Interdiscip. Rev. Syst. Biol. Med.* 1 380–389. 10.1002/wsbm.22 20836003

[B50] Cabrera, DeBuc D. Somfai G. M. Arthur E. Kostic M. Oropesa S. (2018). Investigating multimodal diagnostic eye biomarkers of cognitive impairment by measuring vascular and neurogenic changes in the Retina. *Front. Physiol.* 9:1721. 10.3389/fphys.2018.01721 30574092 PMC6291749

[B51] Cai J. Qi X. Kociok N. Skosyrski S. Emilio A. Ruan Q. (2012). beta-Secretase (BACE1) inhibition causes retinal pathology by vascular dysregulation and accumulation of age pigment. *EMBO Mol. Med.* 4 980–991. 10.1002/emmm.201101084 22903875 PMC3491829

[B52] Cardona A. E. Pioro E. P. Sasse M. E. Kostenko V. Cardona S. M. Dijkstra I. M. (2006). Control of microglial neurotoxicity by the fractalkine receptor. *Nat. Neurosci.* 9 917–924. 10.1038/nn1715 16732273

[B53] Carmine-Simmen K. Proctor T. Tschape J. Poeck B. Triphan T. Strauss R. (2009). Neurotoxic effects induced by the Drosophila amyloid-beta peptide suggest a conserved toxic function. *Neurobiol. Dis.* 33 274–281. 10.1016/j.nbd.2008.10.014 19049874 PMC4418460

[B54] Cerquera-Jaramillo M. A. Nava-Mesa M. O. González-Reyes R. E. Tellez-Conti C. De-La-Torre A. (2018). Visual features in Alzheimer’s disease: from basic mechanisms to clinical overview. *Neural Plast.* 2018 2941783. 10.1155/2018/2941783 30405709 PMC6204169

[B55] Cesareo M. Martucci A. Ciuffoletti E. Mancino R. Cerulli A. Sorge R. P. (2015). Association between Alzheimer’s disease and glaucoma: a study based on heidelberg retinal tomography and frequency doubling technology perimetry. *Front. Neurosci.* 9:479. 10.3389/fnins.2015.00479 26733792 PMC4683203

[B56] Chandra A. Valkimadi P. E. Pagano G. Cousins O. Dervenoulas G. Politis M. (2019). Applications of amyloid, tau, and neuroinflammation PET imaging to Alzheimer’s disease and mild cognitive impairment. *Hum. Brain Mapp.* 40 5424–5442. 10.1002/hbm.24782 31520513 PMC6864887

[B57] Chang L. Y. Ardiles A. O. Tapia-Rojas C. Araya J. Inestrosa N. C. Palacios A. G. (2020). Evidence of synaptic and neurochemical remodeling in the retina of aging degus. *Front. Neurosci.* 14:161. 10.3389/fnins.2020.00161 32256305 PMC7095275

[B58] Chang L. Y. Lowe J. Ardiles A. Lim J. Grey A. C. Robertson K. (2014). Alzheimer’s disease in the human eye. Clinical tests that identify ocular and visual information processing deficit as biomarkers. *Alzheimers Dement.* 10 251–261. 10.1016/j.jalz.2013.06.004 24011928

[B59] Chen M.-K. Mecca A. P. Naganawa M. Finnema S. J. Toyonaga T. Lin S.-F. (2018). Assessing synaptic density in alzheimer disease with synaptic vesicle glycoprotein 2A positron emission tomographic imaging. *JAMA Neurol.* 75 1215–1224. 10.1001/jamaneurol.2018.1836 30014145 PMC6233853

[B60] Chen S. K. Badea T. C. Hattar S. (2011). Photoentrainment and pupillary light reflex are mediated by distinct populations of ipRGCs. *Nature* 476 92–95. 10.1038/nature10206 21765429 PMC3150726

[B61] Cheung C. Y. Ong Y. T. Ikram M. K. Ong S. Y. Li X. Hilal S. (2014). Microvascular network alterations in the retina of patients with Alzheimer’s disease. *Alzheimers Dement.* 10 135–142. 10.1016/j.jalz.2013.06.009 24439169

[B62] Chiasseu M. Alarcon-Martinez L. Belforte N. Quintero H. Dotigny F. Destroismaisons L. (2017). Tau accumulation in the retina promotes early neuronal dysfunction and precedes brain pathology in a mouse model of Alzheimer’s disease. *Mol. Neurodegen.* 12:58. 10.1186/s13024-017-0199-3 28774322 PMC5543446

[B63] Choi S. H. Kim Y. H. Hebisch M. Sliwinski C. Lee S. D’avanzo C. (2014). A three-dimensional human neural cell culture model of Alzheimer’s disease. *Nature* 515 274–278. 10.1038/nature13800 25307057 PMC4366007

[B64] Chougule P. S. Najjar R. P. Finkelstein M. T. Kandiah N. Milea D. (2019). Light-induced pupillary responses in Alzheimer’s disease. *Front. Neurol.* 10:360. 10.3389/fneur.2019.00360 31031692 PMC6473037

[B65] Chouhan A. K. Guo C. Hsieh Y. C. Ye H. Senturk M. Zuo Z. (2016). Uncoupling neuronal death and dysfunction in Drosophila models of neurodegenerative disease. *Acta Neuropathol. Commun.* 4:62. 10.1186/s40478-016-0333-4 27338814 PMC4918017

[B66] Colligris P. Perez, De Lara M. J. Colligris B. Pintor J. (2018). Ocular manifestations of Alzheimer’s and other neurodegenerative diseases: the prospect of the eye as a tool for the early diagnosis of Alzheimer’s disease. *J. Ophthalmol.* 2018:8538573. 10.1155/2018/8538573 30151279 PMC6091327

[B67] Cordeiro M. F. Guo L. Luong V. Harding G. Wang W. Jones H. E. (2004). Real-time imaging of single nerve cell apoptosis in retinal neurodegeneration. *Proc. Natl. Acad. Sci. U.S.A.* 101 13352–13356. 10.1073/pnas.0405479101 15340151 PMC516570

[B68] Cormack F. K. Tovee M. Ballard C. (2000). Contrast sensitivity and visual acuity in patients with Alzheimer’s disease. *Int. J. Geriatr. Psychiatry* 15 614–620. 10.1002/1099-1166(200007)15:7<614::AID-GPS153>3.0.CO;2-0 10918342

[B69] Crair M. C. Mason C. A. (2016). Reconnecting eye to brain. *J. Neurosci.* 36 10707–10722. 10.1523/JNEUROSCI.1711-16.2016 27798125 PMC5083002

[B70] Criscuolo C. Cerri E. Fabiani C. Capsoni S. Cattaneo A. Domenici L. (2018). The retina as a window to early dysfunctions of Alzheimer’s disease following studies with a 5xFAD mouse model. *Neurobiol. Aging* 67 181–188. 10.1016/j.neurobiolaging.2018.03.017 29735432

[B71] Cronin-Golomb A. (1995). Vision in Alzheimer’s disease. *Gerontologist* 35 370–376. 10.1093/geront/35.3.370 7622089

[B72] Cronin-Golomb A. Rizzo J. F. Corkin S. Growdon J. H. (1991). Visual function in Alzheimer’s disease and normal aging. *Ann. N. Y. Acad. Sci.* 640 28–35. 10.1111/j.1749-6632.1991.tb00186.x 1776752

[B73] Cronin-Golomb A. Sugiura R. Corkin S. Growdon J. H. (1993). Incomplete achromatopsia in Alzheimer’s disease. *Neurobiol. Aging* 14 471–477. 10.1016/0197-4580(93)90105-K 8247229

[B74] Cubo E. Tedejo R. P. Rodriguez Mendez V. Lopez Pena M. J. Trejo Gabriel Y. G. J. M. (2010). Retina thickness in Parkinson’s disease and essential tremor. *Mov. Disord.* 25 2461–2462. 10.1002/mds.23215 20669291

[B75] Cunha J. P. Proença R. Dias-Santos A. Melancia D. Almeida R. Águas H. (2017). Choroidal thinning: Alzheimer’s disease and aging. *Alzheimers Dement.* 8 11–17. 10.1016/j.dadm.2017.03.004 28435851 PMC5390660

[B76] Cunha L. P. Almeida A. L. Costa-Cunha L. V. Costa C. F. Monteiro M. L. (2016). The role of optical coherence tomography in Alzheimer’s disease. *Int. J. Retina Vitreous* 2:24. 10.1186/s40942-016-0049-4 27847642 PMC5088456

[B77] Cunha-Vaz J. (2017). The blood-retinal barrier in the management of retinal disease: EURETINA award lecture. *Ophthalmologica* 237 1–10. 10.1159/000455809 28152535

[B78] Cunha-Vaz J. Bernardes R. Lobo C. (2011). Blood-retinal barrier. *Eur. J. Ophthalmol.* 21(Suppl. 6), S3–S9. 10.5301/EJO.2010.6049 23264323

[B79] Cunha-Vaz J. Santos T. Alves D. Marques I. Neves C. Soares M. (2017). Agreement between OCT leakage and fluorescein angiography to identify sites of alteration of the blood-retinal barrier in diabetes. *Ophthalmol. Retina* 1 395–403. 10.1016/j.oret.2017.02.002 31047568

[B80] Cunha-Vaz J. Santos T. Ribeiro L. Alves D. Marques I. Goldberg M. (2016). OCT-Leakage: a new method to identify and locate abnormal fluid accumulation in diabetic Retinal Edema. *Invest. Ophthalmol. Vis. Sci.* 57 6776–6783. 10.1167/iovs.16-19999 27978559

[B81] Cutler T. Sarkar A. Moran M. Steffensmeier A. Puli O. R. Mancini G. (2015). Drosophila eye model to study neuroprotective role of CREB binding protein (CBP) in Alzheimer’s disease. *PLoS One* 10:e0137691. 10.1371/journal.pone.0137691 26367392 PMC4569556

[B82] Dacey D. M. Liao H. W. Peterson B. B. Robinson F. R. Smith V. C. Pokorny J. (2005). Melanopsin-expressing ganglion cells in primate retina signal colour and irradiance and project to the LGN. *Nature* 433 749–754. 10.1038/nature03387 15716953

[B83] De Strooper B. Karran E. (2016). The cellular phase of Alzheimer’s disease. *Cell* 164 603–615. 10.1016/j.cell.2015.12.056 26871627

[B84] Deal J. A. Sharrett A. R. Rawlings A. M. Gottesman R. F. Bandeen-Roche K. Albert M. (2018). Retinal signs and 20-year cognitive decline in the atherosclerosis risk in communities study. *Neurology* 90 e1158–e1166. 10.1212/WNL.0000000000005205 29490915 PMC5880633

[B85] Deczkowska A. Keren-Shaul H. Weiner A. Colonna M. Schwartz M. Amit I. (2018). Disease-associated microglia: a universal immune sensor of neurodegeneration. *Cell* 173 1073–1081. 10.1016/j.cell.2018.05.003 29775591

[B86] DeMattos R. B. Bales K. R. Cummins D. J. Paul S. M. Holtzman D. M. (2002). Brain to plasma amyloid-beta efflux: a measure of brain amyloid burden in a mouse model of Alzheimer’s disease. *Science* 295 2264–2267. 10.1126/science.1067568 11910111

[B87] Demb J. B. Singer J. H. (2015). Functional circuitry of the retina. *Annu. Rev. Vis. Sci.* 1 263–289. 10.1146/annurev-vision-082114-035334 28532365 PMC5749398

[B88] den Haan J. Morrema T. H. J. Verbraak F. D. De Boer J. F. Scheltens P. Rozemuller A. J. (2018). Amyloid-beta and phosphorylated tau in post-mortem Alzheimer’s disease retinas. *Acta Neuropathol. Commun.* 6:147. 10.1186/s40478-018-0650-x 30593285 PMC6309096

[B89] DeSimone C. V. Graff-Radford J. El-Harasis M. A. Rabinstein A. A. Asirvatham S. J. Homles D. R. Jr. (2017). Cerebral amyloid angiopathy: diagnosis, clinical implications, and management strategies in atrial fibrillation. *J. Am. Coll. Cardiol.* 70 1173–1182. 10.1016/j.jacc.2017.07.724 28838368

[B90] Do T. M. Dodacki A. Alata W. Calon F. Nicolic S. Scherrmann J. M. (2015). Age-dependent regulation of the blood-brain barrier influx/efflux equilibrium of amyloid-beta peptide in a mouse model of Alzheimer’s disease (3xTg-AD). *J. Alzheimers Dis.* 49 287–300. 10.3233/JAD-150350 26484906

[B91] Do carmo A. Ramos P. Reis A. Proenca R. Cunha-Vaz J. G. (1998). Breakdown of the inner and outer blood retinal barrier in streptozotocin-induced diabetes. *Exp. Eye Res.* 67 569–575. 10.1006/exer.1998.0546 9878219

[B92] Doraiswamy P. M. Sperling R. A. Johnson K. Reiman E. M. Wong T. Z. Sabbagh M. N. (2014). Florbetapir F 18 amyloid PET and 36-month cognitive decline: a prospective multicenter study. *Mol. Psychiatry* 19 1044–1051. 10.1038/mp.2014.9 24614494 PMC4195975

[B93] Douglas W. Scharre M. (2019). Preclinical, prodromal, and dementia stages of Alzheimer’s disease. *Pract. Neurol.* 36–42.30097552

[B94] Doustar J. Torbati T. Black K. L. Koronyo Y. Koronyo-Hamaoui M. (2017). Optical coherence tomography in Alzheimer’s disease and other neurodegenerative diseases. *Front. Neurol.* 8:701. 10.3389/fneur.2017.00701 29312125 PMC5742098

[B95] Du L. Y. Chang L. Y. Ardiles A. O. Tapia-Rojas C. Araya J. Inestrosa N. C. (2015). Alzheimer’s disease-related protein expression in the retina of octodon degus. *PLoS One* 10:e0135499. 10.1371/journal.pone.0135499 26267479 PMC4534194

[B96] Dubois B. (2018). The emergence of a new conceptual framework for Alzheimer’s disease. *J. Alzheimers Dis.* 62 1059–1066. 10.3233/JAD-170536 29036825 PMC5870001

[B97] Dubois B. Chupin M. Hampel H. Lista S. Cavedo E. Croisile B. (2015). Donepezil decreases annual rate of hippocampal atrophy in suspected prodromal Alzheimer’s disease. *Alzheimers Dement.* 11 1041–1049. 10.1016/j.jalz.2014.10.003 25596420

[B98] Dutescu R. M. Li Q. X. Crowston J. Masters C. L. Baird P. N. Culvenor J. G. (2009). Amyloid precursor protein processing and retinal pathology in mouse models of Alzheimer’s disease. *Graefes Arch. Clin. Exp. Ophthalmol.* 247 1213–1221. 10.1007/s00417-009-1060-3 19271231

[B99] Edison P. Donat C. K. Sastre M. (2018). In vivo imaging of glial activation in Alzheimer’s disease. *Front. Neurol.* 9:625. 10.3389/fneur.2018.00625 30131755 PMC6090997

[B100] Edwards M. M. Rodriguez J. J. Gutierrez-Lanza R. Yates J. Verkhratsky A. Lutty G. A. (2014). Retinal macroglia changes in a triple transgenic mouse model of Alzheimer’s disease. *Exp. Eye Res.* 127 252–260. 10.1016/j.exer.2014.08.006 25149907 PMC4175519

[B101] Einarsdottir A. B. Hardarson S. H. Kristjansdottir J. V. Bragason D. T. Snaedal J. Stefansson E. (2016). Retinal oximetry imaging in Alzheimer’s disease. *J. Alzheimers Dis.* 49 79–83. 10.3233/JAD-150457 26444785

[B102] Engelhardt B. Carare R. O. Bechmann I. Flugel A. Laman J. D. Weller R. O. (2016). Vascular, glial, and lymphatic immune gateways of the central nervous system. *Acta Neuropathol.* 132 317–338. 10.1007/s00401-016-1606-5 27522506 PMC4992028

[B103] Erskine L. Herrera E. (2014). Connecting the retina to the brain. *ASN Neuro* 6:1759091414562107. 10.1177/1759091414562107 25504540 PMC4720220

[B104] Feke G. T. Hyman B. T. Stern R. A. Pasquale L. R. (2015). Retinal blood flow in mild cognitive impairment and Alzheimer’s disease. *Alzheimers Dement.* 1 144–151. 10.1016/j.dadm.2015.01.004 27239502 PMC4876882

[B105] Feng R. Li L. Yu H. Liu M. Zhao W. (2016). Melanopsin retinal ganglion cell loss and circadian dysfunction in Alzheimer’s disease (Review). *Mol. Med. Rep.* 13 3397–3400. 10.3892/mmr.2016.4966 26935586 PMC4805057

[B106] Ferrari L. Huang S. C. Magnani G. Ambrosi A. Comi G. Leocani L. (2017). Optical coherence tomography reveals retinal neuroaxonal thinning in frontotemporal dementia as in Alzheimer’s disease. *J. Alzheimers Dis.* 56 1101–1107. 10.3233/JAD-160886 28106555

[B107] Finelli A. Kelkar A. Song H. J. Yang H. Konsolaki M. (2004). A model for studying Alzheimer’s Abeta42-induced toxicity in *Drosophila melanogaster*. *Mol. Cell Neurosci.* 26 365–375. 10.1016/j.mcn.2004.03.001 15234342

[B108] Fiorentini A. Porciatti V. Morrone M. C. Burr D. C. (1996). Visual ageing: unspecific decline of the responses to luminance and colour. *Vision Res.* 36 3557–3566. 10.1016/0042-6989(96)00032-6 8977022

[B109] Fisher J. B. Jacobs D. A. Markowitz C. E. Galetta S. L. Volpe N. J. Nano-Schiavi M. L. (2006). Relation of visual function to retinal nerve fiber layer thickness in multiple sclerosis. *Ophthalmology* 113 324–332. 10.1016/j.ophtha.2005.10.040 16406539

[B110] Frederikse P. H. Ren X. O. (2002). Lens defects and age-related fiber cell degeneration in a mouse model of increased AbetaPP gene dosage in Down syndrome. *Am. J. Pathol.* 161 1985–1990. 10.1016/S0002-9440(10)64475-6 12466113 PMC1850926

[B111] Frenkel D. Maron R. Burt D. S. Weiner H. L. (2005). Nasal vaccination with a proteosome-based adjuvant and glatiramer acetate clears beta-amyloid in a mouse model of Alzheimer disease. *J. Clin. Invest.* 115 2423–2433. 10.1172/JCI23241 16100572 PMC1184038

[B112] Friberg T. R. Lace J. Rosenstock J. Raskin P. (1987). Retinal microaneurysm counts in diabetic retinopathy: colour photography versus fluorescein angiography. *Can. J. Ophthalmol.* 22 226–229. 3607597

[B113] Friker L. L. Scheiblich H. Hochheiser I. V. Brinkschulte R. Riedel D. Latz E. (2020). β-Amyloid clustering around ASC fibrils boosts its toxicity in microglia. *Cell Rep.* 30 3743.e6–3754.e6. 10.1016/j.celrep.2020.02.025 32187546 PMC8729885

[B114] Frost S. Kanagasingam Y. Macaulay S. L. Koronyo-Hamaoui M. Koronyo Y. Biggs D. (2014). Retinal amyloid fluorescence imaging predicts cerebral amyloid burden and Alzheimer’s disease. *Alzheimer’s Dement.* 10 234–235. 10.1016/j.jalz.2014.04.341

[B115] Frost S. Kanagasingam Y. Sohrabi H. Vignarajan J. Bourgeat P. Salvado O. (2013). Retinal vascular biomarkers for early detection and monitoring of Alzheimer’s disease. *Transl. Psychiatry* 3:e233. 10.1038/tp.2012.150 23443359 PMC3591002

[B116] Frost S. Martins R. N. Kanagasingam Y. (2010). Ocular biomarkers for early detection of Alzheimer’s disease. *J. Alzheimers Dis.* 22 1–16. 10.3233/JAD-2010-100819 20847434

[B117] Fuhrmann M. Bittner T. Jung C. K. Burgold S. Page R. M. Mitteregger G. (2010). Microglial Cx3cr1 knockout prevents neuron loss in a mouse model of Alzheimer’s disease. *Nat. Neurosci.* 13 411–413. 10.1038/nn.2511 20305648 PMC4072212

[B118] Gao L. Chen X. Tang Y. Zhao J. Li Q. Fan X. (2015). Neuroprotective effect of memantine on the retinal ganglion cells of APPswe/PS1DeltaE9 mice and its immunomodulatory mechanisms. *Exp. Eye Res.* 135 47–58. 10.1016/j.exer.2015.04.013 25912193

[B119] Garcia-Alloza M. Borrelli L. A. Rozkalne A. Hyman B. T. Bacskai B. J. (2007). Curcumin labels amyloid pathology in vivo, disrupts existing plaques, and partially restores distorted neurites in an Alzheimer mouse model. *J. Neurochem.* 102 1095–1104. 10.1111/j.1471-4159.2007.04613.x 17472706

[B120] Gauthier S. Rosa-Neto P. (2019). Alzheimer’s disease biomarkers. *Pract. Neurol.* 60–62.

[B121] Gharbiya M. Trebbastoni A. Parisi F. Manganiello S. Cruciani F. D’antonio F. (2014). Choroidal thinning as a new finding in Alzheimer’s disease: evidence from enhanced depth imaging spectral domain optical coherence tomography. *J. Alzheimers Dis.* 40 907–917. 10.3233/JAD-132039 24577467

[B122] Ghiso J. Tomidokoro Y. Revesz T. Frangione B. Rostagno A. (2010). Cerebral amyloid angiopathy and Alzheimer’s disease. *Hirosaki Igaku* 61 S111–S124. 21037967 PMC2964669

[B123] Gilmore G. C. Wenk H. E. Naylor L. A. Koss E. (1994). Motion perception and Alzheimer’s disease. *J. Gerontol.* 49 52–57. 10.1093/geronj/49.2.P52 8126359

[B124] Goedert M. (2015). Neurodegeneration. Alzheimer’s and Parkinson’s diseases: the prion concept in relation to assembled Abeta, tau, and alpha-synuclein. *Science* 349:1255555. 10.1126/science.1255555 26250687

[B125] Goedert M. Spillantini M. G. Jakes R. Rutherford D. Crowther R. A. (1989). Multiple isoforms of human microtubule-associated protein tau: sequences and localization in neurofibrillary tangles of Alzheimer’s disease. *Neuron* 3 519–526. 10.1016/0896-6273(89)90210-9 2484340

[B126] Goldstein L. E. Muffat J. A. Cherny R. A. Moir R. D. Ericsson M. H. Huang X. (2003). Cytosolic beta-amyloid deposition and supranuclear cataracts in lenses from people with Alzheimer’s disease. *Lancet* 361 1258–1265. 10.1016/S0140-6736(03)12981-9 12699953

[B127] Gooley J. J. Lu J. Fischer D. Saper C. B. (2003). A broad role for melanopsin in nonvisual photoreception. *J. Neurosci.* 23 7093–7106. 10.1523/JNEUROSCI.23-18-07093.2003 12904470 PMC6740653

[B128] Gota V. S. Maru G. B. Soni T. G. Gandhi T. R. Kochar N. Agarwal M. G. (2010). Safety and pharmacokinetics of a solid lipid curcumin particle formulation in osteosarcoma patients and healthy volunteers. *J. Agric. Food Chem.* 58 2095–2099. 10.1021/jf9024807 20092313

[B129] Grammenoudi S. Kosmidis S. Skoulakis E. M. (2006). Cell type-specific processing of human Tau proteins in Drosophila. *FEBS Lett.* 580 4602–4606. 10.1016/j.febslet.2006.07.045 16875690

[B130] Granholm E. L. Panizzon M. S. Elman J. A. Jak A. J. Hauger R. L. Bondi M. W. (2017). Pupillary responses as a biomarker of early risk for Alzheimer’s disease. *J. Alzheimers Dis.* 56 1419–1428. 10.3233/JAD-161078 28157098 PMC5808562

[B131] Gravina S. A. Ho L. Eckman C. B. Long K. E. Otvos L. Jr. (1995). Amyloid beta protein (A beta) in Alzheimer’s disease brain. Biochemical and immunocytochemical analysis with antibodies specific for forms ending at A beta 40 or A beta 42(43). *J. Biol. Chem.* 270 7013–7016. 10.1074/jbc.270.13.7013 7706234

[B132] Green A. J. Mcquaid S. Hauser S. L. Allen I. V. Lyness R. (2010). Ocular pathology in multiple sclerosis: retinal atrophy and inflammation irrespective of disease duration. *Brain* 133 1591–1601. 10.1093/brain/awq080 20410146 PMC2877904

[B133] Greeve I. Kretzschmar D. Tschape J. A. Beyn A. Brellinger C. Schweizer M. (2004). Age-dependent neurodegeneration and Alzheimer-amyloid plaque formation in transgenic Drosophila. *J. Neurosci.* 24 3899–3906. 10.1523/JNEUROSCI.0283-04.2004 15102905 PMC6729409

[B134] Grimaldi A. Brighi C. Peruzzi G. Ragozzino D. Bonanni V. Limatola C. (2018). Inflammation, neurodegeneration and protein aggregation in the retina as ocular biomarkers for Alzheimer’s disease in the 3xTg-AD mouse model. *Cell Death Dis.* 9:685. 10.1038/s41419-018-0740-5 29880901 PMC5992214

[B135] Grimaldi A. Pediconi N. Oieni F. Pizzarelli R. Rosito M. Giubettini M. (2019). Neuroinflammatory processes, A1 astrocyte activation and protein aggregation in the retina of Alzheimer’s disease patients, possible biomarkers for early diagnosis. *Front. Neurosci.* 13:925. 10.3389/fnins.2019.00925 31551688 PMC6737046

[B136] Guo L. Salt T. E. Luong V. Wood N. Cheung W. Maass A. (2007). Targeting amyloid-beta in glaucoma treatment. *Proc. Natl. Acad. Sci. U.S.A.* 104 13444–13449. 10.1073/pnas.0703707104 17684098 PMC1940230

[B137] Gupta N. Ang L.-C. De Tilly L. N. Bidaisee L. Yücel Y. H. (2006). Human glaucoma and neural degeneration in intracranial optic nerve, lateral geniculate nucleus, and visual cortex. *Br. J. Ophthalmol.* 90 674–678. 10.1136/bjo.2005.086769 16464969 PMC1860237

[B138] Gupta N. Fong J. Ang L. C. Yucel Y. H. (2008). Retinal tau pathology in human glaucomas. *Can. J. Ophthalmol.* 43 53–60. 10.3129/i07-185 18219347

[B139] Gupta V. K. Chitranshi N. Gupta V. B. Golzan M. Dheer Y. Wall R. V. (2016). Amyloid beta accumulation and inner retinal degenerative changes in Alzheimer’s disease transgenic mouse. *Neurosci. Lett.* 623 52–56. 10.1016/j.neulet.2016.04.059 27133194

[B140] Habiba U. Merlin S. Lim J. K. H. Wong V. H. Y. Nguyen C. T. O. Morley J. W. (2020). Age-specific retinal and cerebral immunodetection of Amyloid-β plaques and oligomers in a rodent model of Alzheimer’s disease. *J. Alzheimers Dis*. 76 1135–1150. 10.3233/JAD-191346 32597800

[B141] Hadoux X. Hui F. Lim J. K. H. Masters C. L. Pebay A. Chevalier S. (2019). Non-invasive in vivo hyperspectral imaging of the retina for potential biomarker use in Alzheimer’s disease. *Nat. Commun.* 10:4227. 10.1038/s41467-019-12242-1 31530809 PMC6748929

[B142] Hajee M. E. March W. F. Lazzaro D. R. Wolintz A. H. Shrier E. M. Glazman S. (2009). Inner retinal layer thinning in Parkinson disease. *Arch. Ophthalmol.* 127 737–741. 10.1001/archophthalmol.2009.106 19506190

[B143] Hampel H. Toschi N. Babiloni C. Baldacci F. Black K. L. Bokde A. L. W. (2018). Revolution of Alzheimer precision neurology. passageway of systems biology and neurophysiology. *J. Alzheimers Dis.* 64 S47–S105. 10.3233/JAD-179932 29562524 PMC6008221

[B144] Hanseeuw B. J. Betensky R. A. Jacobs H. I. L. Schultz A. P. Sepulcre J. Becker J. A. (2019). Association of Amyloid and Tau with cognition in preclinical alzheimer disease: a longitudinal study. *JAMA Neurol.* 76 915–924. 10.1001/jamaneurol.2019.1424 31157827 PMC6547132

[B145] Hardy J. Selkoe D. J. (2002). The amyloid hypothesis of Alzheimer’s disease: progress and problems on the road to therapeutics. *Science* 297 353–356. 10.1126/science.1072994 12130773

[B146] Harrison I. F. Whitaker R. Bertelli P. M. O’callaghan J. M. Csincsik L. Bocchetta M. (2019). Optic nerve thinning and neurosensory retinal degeneration in the rTg4510 mouse model of frontotemporal dementia. *Acta Neuropathol. Commun.* 7:4. 10.1186/s40478-018-0654-6 30616676 PMC6322294

[B147] Hart N. J. Koronyo Y. Black K. L. Koronyo-Hamaoui M. (2016). Ocular indicators of Alzheimer’s: exploring disease in the retina. *Acta Neuropathol.* 132 767–787. 10.1007/s00401-016-1613-6 27645291 PMC5106496

[B148] Hattar S. Lucas R. J. Mrosovsky N. Thompson S. Douglas R. H. Hankins M. W. (2003). Melanopsin and rod-cone photoreceptive systems account for all major accessory visual functions in mice. *Nature* 424 76–81. 10.1038/nature01761 12808468 PMC2885907

[B149] Haug B. A. Kolle R. U. Trenkwalder C. Oertel W. H. Paulus W. (1995). Predominant affection of the blue cone pathway in Parkinson’s disease. *Brain* 118(Pt 3), 771–778. 10.1093/brain/118.3.771 7600093

[B150] He Y. Zhao H. Su G. (2014). Ginsenoside Rg1 decreases neurofibrillary tangles accumulation in retina by regulating activities of neprilysin and PKA in retinal cells of AD mice model. *J. Mol. Neurosci.* 52 101–106. 10.1007/s12031-013-0173-7 24287922

[B151] Hellstedt T. Vesti E. Immonen I. (1996). Identification of individual microaneurysms: a comparison between fluorescein angiograms and red-free and colour photographs. *Graefes Arch. Clin. Exp. Ophthalmol.* 234(Suppl. 1), S13–S17. 10.1007/BF02343042 8871144

[B152] Heurling K. Leuzy A. Zimmer E. R. Lubberink M. Nordberg A. (2016). Imaging beta-amyloid using [(18)F]flutemetamol positron emission tomography: from dosimetry to clinical diagnosis. *Eur. J. Nucl. Med. Mol. Imaging* 43 362–373. 10.1007/s00259-015-3208-1 26440450

[B153] Hickman R. A. Faustin A. Wisniewski T. (2016). alzheimer disease and its growing epidemic: risk factors, biomarkers, and the urgent need for therapeutics. *Neurol. Clin.* 34 941–953. 10.1016/j.ncl.2016.06.009 27720002 PMC5116320

[B154] Hinton D. R. Sadun A. A. Blanks J. C. Miller C. A. (1986). Optic-nerve degeneration in Alzheimer’s disease. *N. Engl. J. Med.* 315 485–487. 10.1056/NEJM198608213150804 3736630

[B155] Ho C. Y. Troncoso J. C. Knox D. Stark W. Eberhart C. G. (2014). Beta-amyloid, phospho-tau and alpha-synuclein deposits similar to those in the brain are not identified in the eyes of Alzheimer’s and Parkinson’s disease patients. *Brain Pathol.* 24 25–32. 10.1111/bpa.12070 23714377 PMC3976129

[B156] Hong S. Dissing-Olesen L. Stevens B. (2016). New insights on the role of microglia in synaptic pruning in health and disease. *Curr. Opin. Neurobiol.* 36 128–134. 10.1016/j.conb.2015.12.004 26745839 PMC5479435

[B157] Iadanza M. G. Jackson M. P. Radford S. E. Ranson N. A. (2016). MpUL-multi: software for calculation of amyloid fibril mass per unit length from TB-TEM images. *Sci. Rep.* 6:21078. 10.1038/srep21078 26867957 PMC4751569

[B158] Ibach B. Binder H. Dragon M. Poljansky S. Haen E. Schmitz E. (2006). Cerebrospinal fluid tau and beta-amyloid in Alzheimer patients, disease controls and an age-matched random sample. *Neurobiol. Aging* 27 1202–1211. 10.1016/j.neurobiolaging.2005.06.005 16085339

[B159] Inestrosa N. C. Reyes A. E. Chacon M. A. Cerpa W. Villalon A. Montiel J. (2005). Human-like rodent amyloid-beta-peptide determines Alzheimer pathology in aged wild-type Octodon degu. *Neurobiol. Aging* 26 1023–1028. 10.1016/j.neurobiolaging.2004.09.016 15748782

[B160] Inzelberg R. Ramirez J. A. Nisipeanu P. Ophir A. (2004). Retinal nerve fiber layer thinning in Parkinson disease. *Vision Res.* 44 2793–2797. 10.1016/j.visres.2004.06.009 15342223

[B161] Iqbal K. Liu F. Gong C.-X. Alonso A. D. C. Grundke-Iqbal I. (2009). Mechanisms of tau-induced neurodegeneration. *Acta Neuropathol.* 118 53–69. 10.1007/s00401-009-0486-3 19184068 PMC2872491

[B162] Iseri P. K. Altinas O. Tokay T. Yuksel N. (2006). Relationship between cognitive impairment and retinal morphological and visual functional abnormalities in Alzheimer disease. *J. Neuroophthalmol.* 26 18–24. 10.1097/01.wno.0000204645.56873.26 16518161

[B163] Ising C. Venegas C. Zhang S. Scheiblich H. Schmidt S. V. Vieira-Saecker A. (2019). NLRP3 inflammasome activation drives tau pathology. *Nature* 575 669–673. 10.1038/s41586-019-1769-z 31748742 PMC7324015

[B164] Ivanova E. Alam N. M. Prusky G. T. Sagdullaev B. T. (2019). Blood-retina barrier failure and vision loss in neuron-specific degeneration. *JCI Insight* 4:e126747. 10.1172/jci.insight.126747 30888334 PMC6538333

[B165] Jack C. R. Jr. Bennett D. A. Blennow K. Carrillo M. C. Dunn B. (2018). NIA-AA research framework: toward a biological definition of Alzheimer’s disease. *Alzheimers Dement.* 14 535–562. 10.1016/j.jalz.2018.02.018 29653606 PMC5958625

[B166] James O. G. Doraiswamy P. M. Borges-Neto S. (2015). PET Imaging of Tau pathology in Alzheimer’s disease and tauopathies. *Front. Neurol.* 6:38. 10.3389/fneur.2015.00038 25806018 PMC4353301

[B167] Janez-Escalada L. Janez-Garcia L. Salobrar-Garcia E. Santos-Mayo A. De Hoz R. Yubero R. (2019). Spatial analysis of thickness changes in ten retinal layers of Alzheimer’s disease patients based on optical coherence tomography. *Sci. Rep.* 9:13000. 10.1038/s41598-019-49353-0 31506524 PMC6737098

[B168] Javaid F. Z. Brenton J. Guo L. Cordeiro M. F. (2016). Visual and ocular manifestations of Alzheimer’s disease and their use as biomarkers for diagnosis and progression. *Front. Neurol.* 7:55. 10.3389/fneur.2016.00055 27148157 PMC4836138

[B169] Jentsch S. Schweitzer D. Schmidtke K. U. Peters S. Dawczynski J. Bar K. J. (2015). Retinal fluorescence lifetime imaging ophthalmoscopy measures depend on the severity of Alzheimer’s disease. *Acta Ophthalmol.* 93 e241–e247. 10.1111/aos.12609 25482990

[B170] Jiang H. Wei Y. Shi Y. Wright C. B. Sun X. Gregori G. (2018). Altered macular microvasculature in mild cognitive impairment and Alzheimer disease. *J. Neuroophthalmol.* 38 292–298. 10.1097/WNO.0000000000000580 29040211 PMC5902666

[B171] Jindal V. (2015). Interconnection between brain and retinal neurodegenerations. *Mol. Neurobiol.* 51 885–892. 10.1007/s12035-014-8733-6 24826919

[B172] Johnson J. K. Gross A. L. Pa J. Mclaren D. G. Park L. Q. Manly J. J. (2012). Longitudinal change in neuropsychological performance using latent growth models: a study of mild cognitive impairment. *Brain Imaging Behav.* 6 540–550. 10.1007/s11682-012-9161-8 22562439 PMC3532521

[B173] Johnson K. A. Fox N. C. Sperling R. A. Klunk W. E. (2012). Brain imaging in Alzheimer disease. *Cold Spring Harb. Perspect. Med.* 2:a006213. 10.1101/cshperspect.a006213 22474610 PMC3312396

[B174] Johnson L. V. Leitner W. P. Rivest A. J. Staples M. K. Radeke M. J. Anderson D. H. (2002). The Alzheimer’s A beta-peptide is deposited at sites of complement activation in pathologic deposits associated with aging and age-related macular degeneration. *Proc. Natl. Acad. Sci. U.S.A.* 99 11830–11835. 10.1073/pnas.192203399 12189211 PMC129354

[B175] Ju Y. E. Lucey B. P. Holtzman D. M. (2014). Sleep and Alzheimer disease pathology–a bidirectional relationship. *Nat. Rev. Neurol.* 10 115–119. 10.1038/nrneurol.2013.269 24366271 PMC3979317

[B176] Ju Y. E. Mcleland J. S. Toedebusch C. D. Xiong C. Fagan A. M. Duntley S. P. (2013). Sleep quality and preclinical Alzheimer disease. *JAMA Neurol.* 70 587–593. 10.1001/jamaneurol.2013.2334 23479184 PMC3676720

[B177] Jung N. Y. Han J. C. Ong Y. T. Cheung C. Y. Chen C. P. Wong T. Y. (2019). Retinal microvasculature changes in amyloid-negative subcortical vascular cognitive impairment compared to amyloid-positive Alzheimer’s disease. *J. Neurol. Sci.* 396 94–101. 10.1016/j.jns.2018.10.025 30447606

[B178] Kalia L. V. Lang A. E. (2015). Parkinson’s disease. *Lancet* 386 896–912. 10.1016/S0140-6736(14)61393-3 25904081

[B179] Katz B. Rimmer S. (1989). Ophthalmologic manifestations of Alzheimer’s disease. *Surv. Ophthalmol.* 34 31–43. 10.1016/0039-6257(89)90127-6 2678551

[B180] Kauppinen A. Paterno J. J. Blasiak J. Salminen A. Kaarniranta K. (2016). Inflammation and its role in age-related macular degeneration. *Cell Mol. Life Sci.* 73 1765–1786. 10.1007/s00018-016-2147-8 26852158 PMC4819943

[B181] Kayabasi U. (2018). “Tau in the retina,” in *Procceding of the 21st World Congress on Neurology and Therapeutics*, London.

[B182] Kayabasi U. Sergott R. Rispoli M. (2014). Retinal examination for the diagnosis of Alzheimer’s disease. *Int. J. Ophthal. Pathol.* 1 3–4. 10.4172/2324-8599.1000145

[B183] Keable A. Fenna K. Yuen H. M. Johnston D. A. Smyth N. R. Smith C. (2016). Deposition of amyloid β in the walls of human leptomeningeal arteries in relation to perivascular drainage pathways in cerebral amyloid angiopathy. *Biochim. Biophys. Acta* 1862 1037–1046. 10.1016/j.bbadis.2015.08.024 26327684 PMC4827375

[B184] Kersten H. M. Danesh-Meyer H. V. Kilfoyle D. H. Roxburgh R. H. (2015). Optical coherence tomography findings in Huntington’s disease: a potential biomarker of disease progression. *J. Neurol.* 262 2457–2465. 10.1007/s00415-015-7869-2 26233693

[B185] Kesler A. Vakhapova V. Korczyn A. D. Naftaliev E. Neudorfer M. (2011). Retinal thickness in patients with mild cognitive impairment and Alzheimer’s disease. *Clin. Neurol. Neurosurg.* 113 523–526. 10.1016/j.clineuro.2011.02.014 21454010

[B186] Kim B. J. Grossman M. Song D. Saludades S. Pan W. Dominguez-Perez S. (2019). Persistent and progressive outer retina thinning in frontotemporal degeneration. *Front. Neurosci.* 13:298. 10.3389/fnins.2019.00298 31019447 PMC6459211

[B187] Kim B. J. Irwin D. J. Song D. Daniel E. Leveque J. D. Raquib A. R. (2017). Optical coherence tomography identifies outer retina thinning in frontotemporal degeneration. *Neurology* 89 1604–1611. 10.1212/WNL.0000000000004500 28887373 PMC5634666

[B188] Kimbrough I. F. Robel S. Roberson E. D. Sontheimer H. (2015). Vascular amyloidosis impairs the gliovascular unit in a mouse model of Alzheimer’s disease. *Brain* 138 3716–3733. 10.1093/brain/awv327 26598495 PMC5006220

[B189] Kirbas S. Turkyilmaz K. Anlar O. Tufekci A. Durmus M. (2013). Retinal nerve fiber layer thickness in patients with Alzheimer disease. *J. Neuroophthalmol.* 33 58–61. 10.1097/WNO.0b013e318267fd5f 22918296

[B190] Klein H.-U. Mccabe C. Gjoneska E. Sullivan S. E. Kaskow B. J. Tang A. (2019). Epigenome-wide study uncovers large-scale changes in histone acetylation driven by tau pathology in aging and Alzheimer’s human brains. *Nat. Neurosci.* 22 37–46. 10.1038/s41593-018-0291-1 30559478 PMC6516529

[B191] Knickelbein J. E. Chan C. C. Sen H. N. Ferris F. L. Nussenblatt R. B. (2015). Inflammatory mechanisms of age-related macular degeneration. *Int. Ophthalmol. Clin.* 55 63–78. 10.1097/IIO.0000000000000073 26035762 PMC4472429

[B192] Koronyo Y. Biggs D. Barron E. Boyer D. S. Pearlman J. A. Au W. J. (2017). Retinal amyloid pathology and proof-of-concept imaging trial in Alzheimer’s disease. *JCI Insight* 2:e93621. 10.1172/jci.insight.93621 28814675 PMC5621887

[B193] Koronyo Y. Salumbides B. C. Black K. L. Koronyo-Hamaoui M. (2012). Alzheimer’s disease in the retina: imaging retinal abeta plaques for early diagnosis and therapy assessment. *Neurodegener. Dis.* 10 285–293. 10.1159/000335154 22343730

[B194] Koronyo Y. Salumbides B. C. Sheyn J. Pelissier L. Li S. Ljubimov V. (2015). Therapeutic effects of glatiramer acetate and grafted CD115(+) monocytes in a mouse model of Alzheimer’s disease. *Brain* 138 2399–2422. 10.1093/brain/awv150 26049087 PMC4840949

[B195] Koronyo-Hamaoui M. Doustar J. Oviatt M. Black K. L. Koronyo Y. (2020). “Advances in retinal imaging: retinal amyloid imaging,” in *OCT and Imaging in Central Nervous System Diseases: The Eye as a Window to the Brain*, eds Grzybowski A. Barboni P. (Cham: Springer International Publishing), 83–122. 10.1007/978-3-030-26269-3_6

[B196] Koronyo-Hamaoui M. Ko M. K. Koronyo Y. Azoulay D. Seksenyan A. Kunis G. (2009). Attenuation of AD-like neuropathology by harnessing peripheral immune cells: local elevation of IL-10 and MMP-9. *J. Neurochem.* 111 1409–1424. 10.1111/j.1471-4159.2009.06402.x 19780903

[B197] Koronyo-Hamaoui M. Koronyo Y. Ljubimov A. V. Miller C. A. Ko M. K. Black K. L. (2011). Identification of amyloid plaques in retinas from Alzheimer’s patients and noninvasive in vivo optical imaging of retinal plaques in a mouse model. *Neuroimage* 54(Suppl. 1), S204–S217. 10.1016/j.neuroimage.2010.06.020 20550967 PMC2991559

[B198] Koronyo-Hamaoui M. Sheyn J. Hayden E. Y. Li S. Fuchs D.-T. Regis G. C. (2019). Peripherally derived angiotensin converting enzyme-enhanced macrophages alleviate Alzheimer-related disease. *Brain* 143 336–358. 10.1093/brain/awz364 31794021 PMC6935752

[B199] Kremen W. S. Panizzon M. S. Elman J. A. Granholm E. L. Andreassen O. A. Dale A. M. (2019). Pupillary dilation responses as a midlife indicator of risk for Alzheimer’s disease: association with Alzheimer’s disease polygenic risk. *Neurobiol. Aging* 83 114–121. 10.1016/j.neurobiolaging.2019.09.001 31585363 PMC6931134

[B200] Kromer R. Serbecic N. Hausner L. Froelich L. Aboul-Enein F. Beutelspacher S. C. (2014). Detection of retinal nerve fiber layer defects in Alzheimer’s disease using SD-OCT. *Front. Psychiatry* 5:22. 10.3389/fpsyt.2014.00022 24616709 PMC3934110

[B201] Kunis G. Baruch K. Rosenzweig N. Kertser A. Miller O. Berkutzki T. (2013). IFN-γ-dependent activation of the brain’s choroid plexus for CNS immune surveillance and repair. *Brain* 136 3427–3440. 10.1093/brain/awt259 24088808

[B202] La Morgia C. Ross-Cisneros F. N. Koronyo Y. Hannibal J. Gallassi R. Cantalupo G. (2016). Melanopsin retinal ganglion cell loss in Alzheimer disease. *Ann. Neurol.* 79 90–109. 10.1002/ana.24548 26505992 PMC4737313

[B203] La Morgia C. Ross-Cisneros F. N. Sadun A. A. Carelli V. (2017). Retinal ganglion cells and circadian rhythms in Alzheimer’s disease, Parkinson’s disease, and beyond. *Front. Neurol.* 8:162. 10.3389/fneur.2017.00162 28522986 PMC5415575

[B204] Lakshminarayanan V. Lagrave J. Kean M. L. Dick M. Shankle R. (1996). Vision in dementia: contrast effects. *Neurol. Res.* 18 9–15. 10.1080/01616412.1996.11740369 8714529

[B205] Lebson L. Nash K. Kamath S. Herber D. Carty N. Lee D. C. (2010). Trafficking CD11b-positive blood cells deliver therapeutic genes to the brain of amyloid-depositing transgenic mice. *J. Neurosci.* 30 9651–9658. 10.1523/JNEUROSCI.0329-10.2010 20660248 PMC2929651

[B206] Lee S. Varvel N. H. Konerth M. E. Xu G. Cardona A. E. Ransohoff R. M. (2010). CX3CR1 deficiency alters microglial activation and reduces beta-amyloid deposition in two Alzheimer’s disease mouse models. *Am. J. Pathol.* 177 2549–2562. 10.2353/ajpath.2010.100265 20864679 PMC2966811

[B207] Leger F. Fernagut P.-O. Canron M.-H. Léoni S. Vital C. Tison F. (2011). Protein aggregation in the aging retina. *J. Neuropathol. Exp. Neurol.* 70 63–68. 10.1097/NEN.0b013e31820376cc 21157377

[B208] Li L. Luo J. Chen D. Tong J. B. Zeng L. P. Cao Y. Q. (2016). BACE1 in the retina: a sensitive biomarker for monitoring early pathological changes in Alzheimer’s disease. *Neural Regen. Res.* 11 447–453. 10.4103/1673-5374.179057 27127484 PMC4829010

[B209] Li S. Hayden E. Y. Garcia V. J. Fuchs D.-T. Sheyn J. Daley D. A. (2020). Activated bone marrow-derived macrophages eradicate Alzheimer’s-Related Aβ42 oligomers and protect synapses. *Front. Immunol.* 11:49. 10.3389/fimmu.2020.00049 32082319 PMC7005081

[B210] Liew S. C. Penfold P. L. Provis J. M. Madigan M. C. Billson F. A. (1994). Modulation of MHC class II expression in the absence of lymphocytic infiltrates in Alzheimer’s retinae. *J. Neuropathol. Exp. Neurol.* 53 150–157. 10.1097/00005072-199403000-00006 8120537

[B211] Liu B. Rasool S. Yang Z. Glabe C. G. Schreiber S. S. Ge J. (2009). Amyloid-peptide vaccinations reduce {beta}-amyloid plaques but exacerbate vascular deposition and inflammation in the retina of Alzheimer’s transgenic mice. *Am. J. Pathol.* 175 2099–2110. 10.2353/ajpath.2009.090159 19834067 PMC2774073

[B212] Liu D. Zhang L. Li Z. Zhang X. Wu Y. Yang H. (2015). Thinner changes of the retinal nerve fiber layer in patients with mild cognitive impairment and Alzheimer’s disease. *BMC Neurol.* 15:14. 10.1186/s12883-015-0268-6 25886372 PMC4342899

[B213] Lockhart A. Lamb J. R. Osredkar T. Sue L. I. Joyce J. N. Ye L. (2007). PIB is a non-specific imaging marker of amyloid-beta (Abeta) peptide-related cerebral amyloidosis. *Brain* 130 2607–2615. 10.1093/brain/awm191 17698496

[B214] Loffler K. U. Edward D. P. Tso M. O. (1995). Immunoreactivity against tau, amyloid precursor protein, and beta-amyloid in the human retina. *Invest. Ophthalmol. Vis. Sci.* 36 24–31. 7822152

[B215] London A. Benhar I. Schwartz M. (2013). The retina as a window to the brain-from eye research to CNS disorders. *Nat. Rev. Neurol.* 9 44–53. 10.1038/nrneurol.2012.227 23165340

[B216] Lu J. Shiromani P. Saper C. B. (1999). Retinal input to the sleep-active ventrolateral preoptic nucleus in the rat. *Neuroscience* 93 209–214. 10.1016/S0306-4522(99)00094-9 10430484

[B217] Lu Y. Li Z. Zhang X. Ming B. Jia J. Wang R. (2010). Retinal nerve fiber layer structure abnormalities in early Alzheimer’s disease: evidence in optical coherence tomography. *Neurosci. Lett.* 480 69–72. 10.1016/j.neulet.2010.06.006 20609426

[B218] Luibl V. Isas J. M. Kayed R. Glabe C. G. Langen R. Chen J. (2006). Drusen deposits associated with aging and age-related macular degeneration contain nonfibrillar amyloid oligomers. *J. Clin. Invest.* 116 378–385. 10.1172/JCI25843 16453022 PMC1359048

[B219] Markwell E. L. Feigl B. Zele A. J. (2010). Intrinsically photosensitive melanopsin retinal ganglion cell contributions to the pupillary light reflex and circadian rhythm. *Clin. Exp. Optom.* 93 137–149. 10.1111/j.1444-0938.2010.00479.x 20557555

[B220] Marmor M. F. Ravin J. G. (2011). Fluorescein angiography: insight and serendipity a half century ago. *Arch. Ophthalmol.* 129 943–948. 10.1001/archophthalmol.2011.160 21746986

[B221] Mathis C. A. Mason N. S. Lopresti B. J. Klunk W. E. (2012). Development of positron emission tomography beta-amyloid plaque imaging agents. *Semin. Nucl. Med.* 42 423–432. 10.1053/j.semnuclmed.2012.07.001 23026364 PMC3520098

[B222] Maude R. J. Dondorp A. M. Abu Sayeed A. Day N. P. White N. J. Beare N. A. (2009). The eye in cerebral malaria: what can it teach us? *Trans. R. Soc. Trop. Med. Hyg.* 103 661–664. 10.1016/j.trstmh.2008.11.003 19100590 PMC2700878

[B223] McGrory S. Cameron J. R. Pellegrini E. Warren C. Doubal F. N. Deary I. J. (2017). The application of retinal fundus camera imaging in dementia: a systematic review. *Alzheimers Dement.* 6 91–107. 10.1016/j.dadm.2016.11.001 28229127 PMC5312461

[B224] Meli G. Lecci A. Manca A. Krako N. Albertini V. Benussi L. (2014). Conformational targeting of intracellular Abeta oligomers demonstrates their pathological oligomerization inside the endoplasmic reticulum. *Nat. Commun.* 5:3867. 10.1038/ncomms4867 24861166 PMC4050278

[B225] Melun J. P. Morin L. M. Muise J. G. Desrosiers M. (2001). Color vision deficiencies in Gilles de la Tourette syndrome. *J. Neurol. Sci.* 186 107–110. 10.1016/S0022-510X(01)00516-0 11412879

[B226] Montagne A. Nation D. A. Sagare A. P. Barisano G. Sweeney M. D. Chakhoyan A. (2020). APOE4 leads to blood-brain barrier dysfunction predicting cognitive decline. *Nature* 581 71–76. 10.1038/s41586-020-2247-3 32376954 PMC7250000

[B227] More S. S. Beach J. M. Mcclelland C. Mokhtarzadeh A. Vince R. (2019). In vivo assessment of retinal biomarkers by hyperspectral imaging: early detection of Alzheimer’s disease. *ACS Chem. Neurosci*. 10 4492–4501. 10.1021/acschemneuro.9b00331 31603648

[B228] More S. S. Beach J. M. Vince R. (2016). Early detection of amyloidopathy in Alzheimer’s mice by hyperspectral endoscopy. *Invest. Ophthalmol. Vis. Sci.* 57 3231–3238. 10.1167/iovs.15-17406 27333181

[B229] More S. S. Vince R. (2015). Hyperspectral imaging signatures detect amyloidopathy in Alzheimer’s mouse retina well before onset of cognitive decline. *ACS Chem. Neurosci.* 6 306–315. 10.1021/cn500242z 25354367

[B230] Moreno-Ramos T. Benito-Leon J. Villarejo A. Bermejo-Pareja F. (2013). Retinal nerve fiber layer thinning in dementia associated with Parkinson’s disease, dementia with Lewy bodies, and Alzheimer’s disease. *J. Alzheimers Dis.* 34 659–664. 10.3233/JAD-121975 23271313

[B231] Morin P. J. Abraham C. R. Amaratunga A. Johnson R. J. Huber G. Sandell J. H. (1993). Amyloid precursor protein is synthesized by retinal ganglion cells, rapidly transported to the optic nerve plasma membrane and nerve terminals, and metabolized. *J. Neurochem.* 61 464–473. 10.1111/j.1471-4159.1993.tb02147.x 7687653

[B232] Moschos M. M. Chatziralli I. P. (2017). Evaluation of choroidal and retinal thickness changes in Parkinson’s disease using spectral domain optical coherence tomography. *Semin. Ophthalmol.* 33 494–497. 10.1080/08820538.2017.1307423 28394663

[B233] Moschos M. M. Markopoulos I. Chatziralli I. Rouvas A. Papageorgiou S. G. Ladas I. (2012). Structural and functional impairment of the retina and optic nerve in Alzheimer’s disease. *Curr. Alzheimer Res.* 9 782–788. 10.2174/156720512802455340 22698074

[B234] Moschos M. M. Tagaris G. Markopoulos I. Margetis I. Tsapakis S. Kanakis M. (2011). Morphologic changes and functional retinal impairment in patients with Parkinson disease without visual loss. *Eur. J. Ophthalmol.* 21 24–29. 10.5301/EJO.2010.1318 20602324

[B235] Narayanaswami V. Dahl K. Bernard-Gauthier V. Josephson L. Cumming P. Vasdev N. (2018). Emerging PET radiotracers and targets for imaging of neuroinflammation in neurodegenerative diseases: outlook beyond TSPO. *Mol. Imaging* 17 1536012118792317. 10.1177/1536012118792317 30203712 PMC6134492

[B236] Naskar R. Wissing M. Thanos S. (2002). Detection of early neuron degeneration and accompanying microglial responses in the retina of a rat model of glaucoma. *Invest. Ophthalmol. Vis. Sci.* 43 2962–2968. 12202516

[B237] Ng S. Villemagne V. L. Berlangieri S. Lee S. T. Cherk M. Gong S. J. (2007). Visual assessment versus quantitative assessment of 11C-PIB PET and 18F-FDG PET for detection of Alzheimer’s disease. *J. Nucl. Med.* 48 547–552. 10.2967/jnumed.106.037762 17401090

[B238] Ngoo Q. Z. Wan Hitam W. H. Ab Razak A. (2019). Evaluation of retinal nerve fiber layer thickness, electroretinogram and visual evoked potential in patients with Alzheimer’s disease. *J. Ophthalmol.* 2019:6248185. 10.1155/2019/6248185 31949951 PMC6948353

[B239] Nikolakopoulou A. M. Zhao Z. Montagne A. Zlokovic B. V. (2017). Regional early and progressive loss of brain pericytes but not vascular smooth muscle cells in adult mice with disrupted platelet-derived growth factor receptor-β signaling. *PLoS One* 12:e0176225. 10.1371/journal.pone.0176225 28441414 PMC5404855

[B240] Ning A. Cui J. To E. Ashe K. H. Matsubara J. (2008). Amyloid-beta deposits lead to retinal degeneration in a mouse model of Alzheimer disease. *Invest. Ophthalmol. Vis. Sci.* 49 5136–5143. 10.1167/iovs.08-1849 18566467 PMC3947384

[B241] Nolan J. M. Loskutova E. Howard A. N. Moran R. Mulcahy R. Stack J. (2014). Macular pigment, visual function, and macular disease among subjects with Alzheimer’s disease: an exploratory study. *J. Alzheimers Dis.* 42 1191–1202. 10.3233/JAD-140507 25024317

[B242] O’Bryhim B. E. Apte R. S. Kung N. Coble D. Van Stavern G. P. (2018). Association of preclinical Alzheimer disease with optical coherence tomographic angiography findings. *JAMA Ophthalmol.* 136 1242–1248. 10.1001/jamaophthalmol.2018.3556 30352114 PMC6248182

[B243] Oku H. Kida T. Horie T. Taki K. Mimura M. Kojima S. (2019). Tau is involved in death of retinal ganglion cells of rats from optic nerve crush. *Invest. Ophthalmol. Vis. Sci.* 60 2380–2387. 10.1167/iovs.19-26683 31141609

[B244] Oliveira-Souza F. G. Deramus M. L. Van Groen T. Lambert A. E. Bolding M. S. Strang C. E. (2017). Retinal changes in the Tg-SwDI mouse model of Alzheimer’s disease. *Neuroscience* 354 43–53. 10.1016/j.neuroscience.2017.04.021 28450267 PMC5495115

[B245] Ono M. Saji H. (2011). SPECT imaging agents for detecting cerebral beta-amyloid plaques. *Int. J. Mol. Imaging* 2011:543267. 10.1155/2011/543267 21603239 PMC3094870

[B246] Ontiveros-Torres M. A. Labra-Barrios M. L. Diaz-Cintra S. Aguilar-Vazquez A. R. Moreno-Campuzano S. Flores-Rodriguez P. (2016). Fibrillar amyloid-beta accumulation triggers an inflammatory mechanism leading to hyperphosphorylation of the carboxyl-terminal end of Tau polypeptide in the hippocampal formation of the 3xTg-AD transgenic mouse. *J. Alzheimers Dis.* 52 243–269. 10.3233/JAD-150837 27031470

[B247] Pache M. Smeets C. H. Gasio P. F. Savaskan E. Flammer J. Wirz-Justice A. (2003). Colour vision deficiencies in Alzheimer’s disease. *Age Age.* 32 422–426. 10.1093/ageing/32.4.422 12851187

[B248] Paquet C. Boissonnot M. Roger F. Dighiero P. Gil R. Hugon J. (2007). Abnormal retinal thickness in patients with mild cognitive impairment and Alzheimer’s disease. *Neurosci. Lett.* 420 97–99. 10.1016/j.neulet.2007.02.090 17543991

[B249] Parisi V. Restuccia R. Fattapposta F. Mina C. Bucci M. G. Pierelli F. (2001). Morphological and functional retinal impairment in Alzheimer’s disease patients. *Clin. Neurophysiol.* 112 1860–1867. 10.1016/S1388-2457(01)00620-4 11595144

[B250] Park S. W. Kim J. H. Mook-Jung I. Kim K. W. Park W. J. Park K. H. (2014). Intracellular amyloid beta alters the tight junction of retinal pigment epithelium in 5XFAD mice. *Neurobiol. Aging* 35 2013–2020. 10.1016/j.neurobiolaging.2014.03.008 24709310

[B251] Parthasarathy R. Chow K. M. Derafshi Z. Fautsch M. P. Hetling J. R. Rodgers D. W. (2015). Reduction of amyloid-beta levels in mouse eye tissues by intra-vitreally delivered neprilysin. *Exp. Eye Res.* 138 134–144. 10.1016/j.exer.2015.06.027 26142956 PMC4659645

[B252] Patterson C. (2018). *World Alzheimer Report 2018: The State of the Art of Dementia Research: New Frontiers.* London: Alzheimer’s Disease International (ADI).

[B253] Patton N. Aslam T. Macgillivray T. Pattie A. Deary I. J. Dhillon B. (2005). Retinal vascular image analysis as a potential screening tool for cerebrovascular disease: a rationale based on homology between cerebral and retinal microvasculatures. *J. Anat.* 206 319–348. 10.1111/j.1469-7580.2005.00395.x 15817102 PMC1571489

[B254] Paulus W. Schwarz G. Werner A. Lange H. Bayer A. Hofschuster M. (1993). Impairment of retinal increment thresholds in Huntington’s disease. *Ann. Neurol.* 34 574–578. 10.1002/ana.410340411 8215245

[B255] Perez S. E. Lumayag S. Kovacs B. Mufson E. J. Xu S. (2009). Beta-amyloid deposition and functional impairment in the retina of the APPswe/PS1DeltaE9 transgenic mouse model of Alzheimer’s disease. *Invest. Ophthalmol. Vis. Sci.* 50 793–800. 10.1167/iovs.08-2384 18791173 PMC3697019

[B256] Perrin R. J. Fagan A. M. Holtzman D. M. (2009). Multimodal techniques for diagnosis and prognosis of Alzheimer’s disease. *Nature* 461 916–922. 10.1038/nature08538 19829371 PMC2810658

[B257] Peter-Derex L. Yammine P. Bastuji H. Croisile B. (2015). Sleep and Alzheimer’s disease. *Sleep Med. Rev.* 19 29–38. 10.1016/j.smrv.2014.03.007 24846773

[B258] Petersen R. C. Smith G. E. Waring S. C. Ivnik R. J. Tangalos E. G. Kokmen E. (1999). Mild cognitive impairment: clinical characterization and outcome. *Arch. Neurol.* 56 303–308. 10.1001/archneur.56.3.303 10190820

[B259] Petit D. Gagnon J. F. Fantini M. L. Ferini-Strambi L. Montplaisir J. (2004). Sleep and quantitative EEG in neurodegenerative disorders. *J. Psychosom. Res.* 56 487–496. 10.1016/j.jpsychores.2004.02.001 15172204

[B260] Pogue A. I. Dua P. Hill J. M. Lukiw W. J. (2015). Progressive inflammatory pathology in the retina of aluminum-fed 5xFAD transgenic mice. *J. Inorg. Biochem*. 152 206–209. 10.1016/j.jinorgbio.2015.07.009 26213226 PMC4681626

[B261] Polans J. Keller B. Carrasco-Zevallos O. M. Larocca F. Cole E. Whitson H. E. (2017). Wide-field retinal optical coherence tomography with wavefront sensorless adaptive optics for enhanced imaging of targeted regions. *Biomed. Opt. Express* 8 16–37. 10.1364/BOE.8.000016 28101398 PMC5231289

[B262] Polo V. Rodrigo M. J. Garcia-Martin E. Otin S. Larrosa J. M. Fuertes M. I. (2017). Visual dysfunction and its correlation with retinal changes in patients with Alzheimer’s disease. *Eye* 31 1034–1041. 10.1038/eye.2017.23 28282060 PMC5519267

[B263] Purves D. (ed.) (2001). “The Retina Chapters 11 and 12,” in *Neuroscience*, 2nd Edn, (Sunderland, MA: Sinauer Associates).

[B264] Purves D. Fitzpatrick D. (2001). *The Retina.* Sunderland, MA: Sinauer Associates.

[B265] Querques G. Borrelli E. Sacconi R. De Vitis L. Leocani L. Santangelo R. (2019). Functional and morphological changes of the retinal vessels in Alzheimer’s disease and mild cognitive impairment. *Sci. Rep.* 9:63. 10.1038/s41598-018-37271-6 30635610 PMC6329813

[B266] Quigley H. A. (1999). Neuronal death in glaucoma. *Prog. Retin. Eye Res.* 18 39–57. 10.1016/S1350-9462(98)00014-7 9920498

[B267] Rabinovici G. D. Furst A. J. O’neil J. P. Racine C. A. Mormino E. C. Baker S. L. (2007). 11C-PIB PET imaging in Alzheimer disease and frontotemporal lobar degeneration. *Neurology* 68 1205–1212. 10.1212/01.wnl.0000259035.98480.ed 17420404

[B268] Rentsendorj A. Sheyn J. Fuchs D. T. Daley D. Salumbides B. C. Schubloom H. E. (2018). A novel role for osteopontin in macrophage-mediated amyloid-beta clearance in Alzheimer’s models. *Brain Behav. Immun.* 67 163–180. 10.1016/j.bbi.2017.08.019 28860067 PMC5865478

[B269] Risacher S. L. Wudunn D. Pepin S. M. Magee T. R. Mcdonald B. C. Flashman L. A. (2013). Visual contrast sensitivity in Alzheimer’s disease, mild cognitive impairment, and older adults with cognitive complaints. *Neurobiol. Aging* 34 1133–1144. 10.1016/j.neurobiolaging.2012.08.007 23084085 PMC3545045

[B270] Risacher S. L. Wudunn D. Tallman E. F. West J. D. Gao S. Farlow M. R. (2020). Visual contrast sensitivity is associated with the presence of cerebral amyloid and tau deposition. *Brain Commun.* 2:fcaa019. 10.1093/braincomms/fcaa019 32309804 PMC7151662

[B271] Rizzo M. Anderson S. W. Dawson J. Nawrot M. (2000). Vision and cognition in Alzheimer’s disease. *Neuropsychologia* 38 1157–1169. 10.1016/S0028-3932(00)00023-3 10838150

[B272] Rodnitzky R. L. (1998). Visual dysfunction in Parkinson’s disease. *Clin. Neurosci.* 5 102–106. 10785835

[B273] Roher A. E. Lowenson J. D. Clarke S. Woods A. S. Cotter R. J. Gowing E. (1993). beta-Amyloid-(1-42) is a major component of cerebrovascular amyloid deposits: implications for the pathology of Alzheimer disease. *Proc. Natl. Acad. Sci. U.S.A.* 90 10836–10840. 10.1073/pnas.90.22.10836 8248178 PMC47873

[B274] Rosenzweig N. Dvir-Szternfeld R. Tsitsou-Kampeli A. Keren-Shaul H. Ben-Yehuda H. Weill-Raynal P. (2019). PD-1/PD-L1 checkpoint blockade harnesses monocyte-derived macrophages to combat cognitive impairment in a tauopathy mouse model. *Nat. Commun.* 10:465. 10.1038/s41467-019-08352-5 30692527 PMC6349941

[B275] Rubsam A. Parikh S. Fort P. E. (2018). Role of inflammation in diabetic retinopathy. *Int. J. Mol. Sci.* 19:942. 10.3390/ijms19040942 29565290 PMC5979417

[B276] Sadun A. A. Bassi C. J. (1990). Optic nerve damage in Alzheimer’s disease. *Ophthalmology* 97 9–17. 10.1016/S0161-6420(90)32621-0 2314849

[B277] Sadun A. A. Borchert M. Devita E. Hinton D. R. Bassi C. J. (1987). Assessment of visual impairment in patients with Alzheimer’s disease. *Am. J. Ophthalmol.* 104 113–120. 10.1016/0002-9394(87)90001-8 3618708

[B278] Salamone G. Di Lorenzo C. Mosti S. Lupo F. Cravello L. Palmer K. (2009). Color discrimination performance in patients with Alzheimer’s disease. *Dement. Geriatr. Cogn. Disord.* 27 501–507. 10.1159/000218366 19451717

[B279] Salobrar-Garcia E. De Hoz R. Ramirez A. I. Lopez-Cuenca I. Rojas P. Vazirani R. (2019). Changes in visual function and retinal structure in the progression of Alzheimer’s disease. *PLoS One* 14:e0220535. 10.1371/journal.pone.0220535 31415594 PMC6695171

[B280] Salobrar-Garcia E. De Hoz R. Rojas B. Ramirez A. I. Salazar J. J. Yubero R. (2015). Ophthalmologic psychophysical tests support OCT findings in mild Alzheimer’s disease. *J. Ophthalmol.* 2015:736949. 10.1155/2015/736949 26106485 PMC4461784

[B281] Salobrar-Garcia E. Rodrigues-Neves A. C. Ramirez A. I. De Hoz R. Fernandez-Albarral J. A. Lopez-Cuenca I. (2020). Microglial activation in the retina of a triple-transgenic Alzheimer’s disease mouse model (3xTg-AD). *Int. J. Mol. Sci.* 21:816. 10.3390/ijms21030816 32012676 PMC7038053

[B282] Salter M. W. Stevens B. (2017). Microglia emerge as central players in brain disease. *Nat. Med.* 23 1018–1027. 10.1038/nm.4397 28886007

[B283] Satue M. Garcia-Martin E. Fuertes I. Otin S. Alarcia R. Herrero R. (2013). Use of Fourier-domain OCT to detect retinal nerve fiber layer degeneration in Parkinson’s disease patients. *Eye* 27 507–514. 10.1038/eye.2013.4 23429414 PMC3626016

[B284] Satue M. Rodrigo M. J. Obis J. Vilades E. Gracia H. Otin S. (2017). Evaluation of progressive visual dysfunction and retinal degeneration in patients with Parkinson’s disease. *Invest. Ophthalmol. Vis. Sci.* 58 1151–1157. 10.1167/iovs.16-20460 28208185

[B285] Schallek J. Geng Y. Nguyen H. Williams D. R. (2013). Morphology and topography of retinal pericytes in the living mouse retina using in vivo adaptive optics imaging and ex vivo characterization. *Invest. Ophthalmol. Vis. Sci.* 54 8237–8250. 10.1167/iovs.13-12581 24150762 PMC3869420

[B286] Schilling L. P. Zimmer E. R. Shin M. Leuzy A. Pascoal T. A. Benedet A. L. (2016). Imaging Alzheimer’s disease pathophysiology with PET. *Dement. Neuropsychol.* 10 79–90. 10.1590/S1980-5764-2016DN1002003 29213438 PMC5642398

[B287] Schmid S. Guenther E. Kohler K. (1995). Changes in Thy-1 antigen immunoreactivity in the rat retina during pre- and postnatal development. *Neurosci. Lett.* 199 91–94. 10.1016/0304-3940(95)12020-5 8584251

[B288] Schön C. Hoffmann N. A. Ochs S. M. Burgold S. Filser S. Steinbach S. (2012). Long-term in vivo imaging of fibrillar tau in the retina of P301S transgenic mice. *PLoS One* 7:e53547. 10.1371/journal.pone.0053547 23300938 PMC3534024

[B289] Schultz N. Byman E. Wennström M. (2020). Levels of retinal amyloid-β correlate with levels of retinal IAPP and hippocampal amyloid-β in neuropathologically evaluated individuals. *J. Alzheimers Dis.* 73 1201–1209. 10.3233/JAD-190868 31884473 PMC7081096

[B290] Schwartz M. Peralta Ramos J. M. Ben-Yehuda H. (2020). A 20-year journey from axonal injury to neurodegenerative diseases and the prospect of immunotherapy for combating Alzheimer’s disease. *J. Immunol.* 204 243–250. 10.4049/jimmunol.1900844 31907265

[B291] Selkoe D. J. (2004). Cell biology of protein misfolding: the examples of Alzheimer’s and Parkinson’s diseases. *Nat. Cell Biol.* 6 1054–1061. 10.1038/ncb1104-1054 15516999

[B292] Selkoe D. J. (2008). Soluble oligomers of the amyloid beta-protein impair synaptic plasticity and behavior. *Behav. Brain Res.* 192 106–113. 10.1016/j.bbr.2008.02.016 18359102 PMC2601528

[B293] Semeraro F. Cancarini A. Dell’omo R. Rezzola S. Romano M. R. Costagliola C. (2015). Diabetic retinopathy: vascular and inflammatory disease. *J. Diabetes Res.* 2015:582060. 10.1155/2015/582060 26137497 PMC4475523

[B294] Sharafi S. M. Sylvestre J. P. Chevrefils C. Soucy J. P. Beaulieu S. Pascoal T. A. (2019). Vascular retinal biomarkers improves the detection of the likely cerebral amyloid status from hyperspectral retinal images. *Alzheimers Dement.* 5 610–617. 10.1016/j.trci.2019.09.006 31650017 PMC6804547

[B295] Shi H. Koronyo Y. Rentsendorj A. Regis G. C. Sheyn J. Fuchs D. T. (2020). Identification of early pericyte loss and vascular amyloidosis in Alzheimer’s disease retina. *Acta Neuropathol*. 139 813–836. 10.1007/s00401-020-02134-w 32043162 PMC7181564

[B296] Shi Z. Wu Y. Wang M. Cao J. Feng W. Cheng Y. (2014). Greater attenuation of retinal nerve fiber layer thickness in Alzheimer’s disease patients. *J. Alzheimers Dis.* 40 277–283. 10.3233/JAD-131898 24413621

[B297] Shrier E. M. Adam C. R. Spund B. Glazman S. Bodis-Wollner I. (2012). Interocular asymmetry of foveal thickness in Parkinson disease. *J. Ophthalmol.* 2012 728457. 10.1155/2012/728457 22900149 PMC3415246

[B298] Sidiqi A. Wahl D. Lee S. Ma D. (2020). In vivo retinal fluorescence imaging with curcumin in an Alzheimer mouse model. *Front. Neurosci.* 14:713. 10.3389/fnins.2020.00713 32719582 PMC7350785

[B299] Simard A. R. Soulet D. Gowing G. Julien J. P. Rivest S. (2006). Bone marrow-derived microglia play a critical role in restricting senile plaque formation in Alzheimer’s disease. *Neuron* 49 489–502. 10.1016/j.neuron.2006.01.022 16476660

[B300] Smith M. Z. Nagy Z. Esiri M. M. (1999). Cell cycle-related protein expression in vascular dementia and Alzheimer’s disease. *Neurosci. Lett.* 271 45–48. 10.1016/S0304-3940(99)00509-1 10471210

[B301] Snyder P. J. Johnson L. N. Lim Y. Y. Santos C. Y. Alber J. Maruff P. (2016). Nonvascular retinal imaging markers of preclinical Alzheimer’s disease. *Alzheimers Dement.* 4 169–178. 10.1016/j.dadm.2016.09.001 27830174 PMC5078641

[B302] Steuer H. Jaworski A. Elger B. Kaussmann M. Keldenich J. Schneider H. (2005). Functional characterization and comparison of the outer blood-retina barrier and the blood-brain barrier. *Invest. Ophthalmol. Vis. Sci.* 46 1047–1053. 10.1167/iovs.04-0925 15728564

[B303] Stevens B. Allen N. J. Vazquez L. E. Howell G. R. Christopherson K. S. Nouri N. (2007). The classical complement cascade mediates CNS synapse elimination. *Cell* 131 1164–1178. 10.1016/j.cell.2007.10.036 18083105

[B304] Sweeney M. D. Sagare A. P. Zlokovic B. V. (2018). Blood-brain barrier breakdown in Alzheimer disease and other neurodegenerative disorders. *Nat. Rev. Neurol.* 14 133–150. 10.1038/nrneurol.2017.188 29377008 PMC5829048

[B305] Takeda S. Wegmann S. Cho H. Devos S. L. Commins C. Roe A. D. (2015). Neuronal uptake and propagation of a rare phosphorylated high-molecular-weight tau derived from Alzheimer’s disease brain. *Nat. Commun.* 6:8490. 10.1038/ncomms9490 26458742 PMC4608380

[B306] Tamura H. Kawakami H. Kanamoto T. Kato T. Yokoyama T. Sasaki K. (2006). High frequency of open-angle glaucoma in Japanese patients with Alzheimer’s disease. *J. Neurol. Sci.* 246 79–83. 10.1016/j.jns.2006.02.009 16564058

[B307] Tang J. Kern T. S. (2011). Inflammation in diabetic retinopathy. *Prog. Retin. Eye Res.* 30 343–358. 10.1016/j.preteyeres.2011.05.002 21635964 PMC3433044

[B308] Tezel G. (2011). The immune response in glaucoma: a perspective on the roles of oxidative stress. *Exp. Eye Res.* 93 178–186. 10.1016/j.exer.2010.07.009 20709058 PMC2998544

[B309] Tian J. Shi J. Mann D. M. (2004). Cerebral amyloid angiopathy and dementia. *Panminerva Med.* 46 253–264. 15876981

[B310] Tian T. Zhang B. Jia Y. Li Z. (2014). Promise and challenge: the lens model as a biomarker for early diagnosis of Alzheimer’s disease. *Dis. Markers* 2014 826503. 10.1155/2014/826503 24688166 PMC3945026

[B311] Tiepolt S. Hesse S. Patt M. Luthardt J. Schroeter M. L. Hoffmann K. T. (2016). Early [F]florbetaben and [C]PiB PET images are a surrogate biomarker of neuronal injury in Alzheimer’s disease. *Eur. J. Nucl. Med. Mol. Imaging* 43 1700–1709. 10.1007/s00259-016-3353-1 27026271

[B312] Trick G. L. Barris M. C. Bickler-Bluth M. (1989). Abnormal pattern electroretinograms in patients with senile dementia of the Alzheimer type. *Ann. Neurol.* 26 226–231. 10.1002/ana.410260208 2774510

[B313] Trick G. L. Trick L. R. Morris P. Wolf M. (1995). Visual field loss in senile dementia of the Alzheimer’s type. *Neurology* 45 68–74. 10.1212/WNL.45.1.68 7824139

[B314] Trost A. Lange S. Schroedl F. Bruckner D. Motloch K. A. Bogner B. (2016). Brain and retinal pericytes: origin, function and role. *Front. Cell. Neurosci.* 10:20. 10.3389/fncel.2016.00020 26869887 PMC4740376

[B315] Tsai C. S. Ritch R. Schwartz B. Lee S. S. Miller N. R. Chi T. (1991). Optic nerve head and nerve fiber layer in Alzheimer’s disease. *Arch. Ophthalmol.* 109 199–204. 10.1001/archopht.1991.01080020045040 1993028

[B316] Tsai Y. Lu B. Ljubimov A. V. Girman S. Ross-Cisneros F. N. Sadun A. A. (2014). Ocular changes in TgF344-AD rat model of Alzheimer’s disease. *Invest. Ophthalmol. Vis. Sci.* 55 523–534. 10.1167/iovs.13-12888 24398104 PMC3907137

[B317] Tucker R. P. Matus A. I. (1988). Microtubule-associated proteins characteristic of embryonic brain are found in the adult mammalian retina. *Dev. Biol.* 130 423–434. 10.1016/0012-1606(88)90338-7 3058539

[B318] Vecino E. Rodriguez F. D. Ruzafa N. Pereiro X. Sharma S. C. (2016). Glia-neuron interactions in the mammalian retina. *Prog. Retin. Eye Res.* 51 1–40. 10.1016/j.preteyeres.2015.06.003 26113209

[B319] Vidal D. Mavet S. (1989). In vitro and in vivo toxicity of T-2 toxin, a Fusarium mycotoxin, to mouse peritoneal macrophages. *Infect. Immun.* 57 2260–2264. 10.1128/IAI.57.7.2260-2264.1989 2499548 PMC313871

[B320] Vidoni E. D. Yeh H. W. Morris J. K. Newell K. L. Alqahtani A. Burns N. C. (2016). Cerebral β-Amyloid angiopathy is associated with earlier dementia onset in Alzheimer’s disease. *Neurodegener. Dis.* 16 218–224. 10.1159/000441919 26756746 PMC4915344

[B321] Villeda S. A. Plambeck K. E. Middeldorp J. Castellano J. M. Mosher K. I. Luo J. (2014). Young blood reverses age-related impairments in cognitive function and synaptic plasticity in mice. *Nat. Med.* 20 659–663. 10.1038/nm.3569 24793238 PMC4224436

[B322] Vinters H. V. (1987). Cerebral amyloid angiopathy. A critical review. *Stroke* 18 311–324. 10.1161/01.STR.18.2.311 3551211

[B323] Vinters H. V. Gilbert J. J. (1983). Cerebral amyloid angiopathy: incidence and complications in the aging brain. II. The distribution of amyloid vascular changes. *Stroke* 14 924–928. 10.1161/01.STR.14.6.924 6658996

[B324] Viswanathan A. Greenberg S. M. (2011). Cerebral amyloid angiopathy in the elderly. *Ann. Neurol.* 70 871–880. 10.1002/ana.22516 22190361 PMC4004372

[B325] Vohra R. Tsai J. C. Kolko M. (2013). The role of inflammation in the pathogenesis of glaucoma. *Surv. Ophthalmol.* 58 311–320. 10.1016/j.survophthal.2012.08.010 23768921

[B326] Vonsattel J. P. Myers R. H. Hedley-Whyte E. T. Ropper A. H. Bird E. D. Richardson E. P. Jr. (1991). Cerebral amyloid angiopathy without and with cerebral hemorrhages: a comparative histological study. *Ann. Neurol.* 30 637–649. 10.1002/ana.410300503 1763890

[B327] Wang C. Holtzman D. M. (2020). Bidirectional relationship between sleep and Alzheimer’s disease: role of amyloid, tau, and other factors. *Neuropsychopharmacology* 45 104–120. 10.1038/s41386-019-0478-5 31408876 PMC6879647

[B328] Wang X. Lou N. Eberhardt A. Yang Y. Kusk P. Xu Q. (2020). An ocular glymphatic clearance system removes β-amyloid from the rodent eye. *Sci. Transl. Med.* 12:eaaw3210. 10.1126/scitranslmed.aaw3210 32213628 PMC7356596

[B329] Weil R. S. Schrag A. E. Warren J. D. Crutch S. J. Lees A. J. Morris H. R. (2016). Visual dysfunction in Parkinson’s disease. *Brain* 139 2827–2843. 10.1093/brain/aww175 27412389 PMC5091042

[B330] Weissová K. Bartoš A. Sládek M. Nováková M. Sumová A. (2016). Moderate changes in the circadian system of Alzheimer’s disease patients detected in their home environment. *PLoS One* 11:e0146200. 10.1371/journal.pone.0146200 26727258 PMC4701009

[B331] Welge V. Fiege O. Lewczuk P. Mollenhauer B. Esselmann H. Klafki H. W. (2009). Combined CSF tau, p-tau181 and amyloid-beta 38/40/42 for diagnosing Alzheimer’s disease. *J. Neural Transm.* 116 203–212. 10.1007/s00702-008-0177-6 19142572

[B332] Werry E. L. Bright F. M. Piguet O. Ittner L. M. Halliday G. M. Hodges J. R. (2019). Recent developments in TSPO PET imaging as a biomarker of neuroinflammation in neurodegenerative disorders. *Int. J. Mol. Sci.* 20:3161. 10.3390/ijms20133161 31261683 PMC6650818

[B333] Wijk H. Berg S. Sivik L. Steen B. (1999). Colour discrimination, colour naming and colour preferences among individuals with Alzheimer’s disease. *Int. J. Geriatr. Psychiatry* 14 1000–1005. 10.1002/(SICI)1099-1166(199912)14:12<1000::AID-GPS46>3.0.CO;2-E 10607966

[B334] Williams E. A. Mcguone D. Frosch M. P. Hyman B. T. Laver N. Stemmer-Rachamimov A. (2017). Absence of Alzheimer disease neuropathologic changes in eyes of subjects with Alzheimer disease. *J. Neuropathol. Exp. Neurol.* 76 376–383. 10.1093/jnen/nlx020 28379416 PMC7191616

[B335] Williams M. A. Mcgowan A. J. Cardwell C. R. Cheung C. Y. Craig D. Passmore P. (2015). Retinal microvascular network attenuation in Alzheimer’s disease. *Alzheimers Dement.* 1 229–235. 10.1016/j.dadm.2015.04.001 26634224 PMC4629099

[B336] Williams P. A. Thirgood R. A. Oliphant H. Frizzati A. Littlewood E. Votruba M. (2013). Retinal ganglion cell dendritic degeneration in a mouse model of Alzheimer’s disease. *Neurobiol. Aging* 34 1799–1806. 10.1016/j.neurobiolaging.2013.01.006 23465714

[B337] Wyss-Coray T. (2006). Inflammation in Alzheimer disease: driving force, bystander or beneficial response? *Nat. Med.* 12 1005–1015. 16960575 10.1038/nm1484

[B338] Wyss-Coray T. Rogers J. (2012). Inflammation in Alzheimer disease-a brief review of the basic science and clinical literature. *Cold Spring Harb. Perspect. Med.* 2:a006346. 10.1101/cshperspect.a006346 22315714 PMC3253025

[B339] Xu Q. Qaum T. Adamis A. P. (2001). Sensitive blood-retinal barrier breakdown quantitation using Evans blue. *Invest. Ophthalmol. Vis. Sci.* 42 789–794. 11222542

[B340] Yang Y. Shiao C. Hemingway J. F. Jorstad N. L. Shalloway B. R. Chang R. (2013). Suppressed retinal degeneration in aged wild type and APPswe/PS1DeltaE9 mice by bone marrow transplantation. *PLoS One* 8:e64246. 10.1371/journal.pone.0064246 23750207 PMC3672108

[B341] Zhao H. Chang R. Che H. Wang J. Yang L. Fang W. (2013). Hyperphosphorylation of tau protein by calpain regulation in retina of Alzheimer’s disease transgenic mouse. *Neurosci. Lett.* 551 12–16. 10.1016/j.neulet.2013.06.026 23810804

[B342] Zhao Z. Sagare A. P. Ma Q. Halliday M. R. Kong P. Kisler K. (2015). Central role for PICALM in amyloid-beta blood-brain barrier transcytosis and clearance. *Nat. Neurosci.* 18 978–987. 10.1038/nn.4025 26005850 PMC4482781

[B343] Zhou R. Caspi R. R. (2010). Ocular immune privilege. *F1000 Biol. Rep.* 2:3. 10.3410/B2-3 20948803 PMC2948372

[B344] Zlokovic B. V. Ghiso J. Mackic J. B. Mccomb J. G. Weiss M. H. Frangione B. (1993). Blood-brain barrier transport of circulating Alzheimer’s amyloid beta. *Biochem. Biophys. Res. Commun.* 197 1034–1040. 10.1006/bbrc.1993.2582 8280117

[B345] Zuroff L. Daley D. Black K. L. Koronyo-Hamaoui M. (2017). Clearance of cerebral Abeta in Alzheimer’s disease: reassessing the role of microglia and monocytes. *Cell Mol. Life Sci.* 74 2167–2201. 10.1007/s00018-017-2463-7 28197669 PMC5425508

